# Settling taxonomic and nomenclatural problems in brine shrimps, *Artemia* (Crustacea: Branchiopoda: Anostraca), by integrating mitogenomics, marker discordances and nomenclature rules

**DOI:** 10.7717/peerj.10865

**Published:** 2021-03-10

**Authors:** Lucía Sainz-Escudero, E. Karen López-Estrada, Paula Carolina Rodríguez-Flores, Mario García-París

**Affiliations:** 1 Museo Nacional de Ciencias Naturales (MNCN-CSIC), Madrid, Spain; 2 Fundación Global Nature, Las Rozas, Madrid, Spain; 3 Centre d’Estudis Avançats de Blanes (CEAB-CSIC), Blanes, Girona, Spain

**Keywords:** Systematics, Phylogeny, New synonymies, Crustacea, Salterns, Salt Lakes, Fossil dating, Mitogenomics

## Abstract

High morphological plasticity in populations of brine shrimp subjected to different environmental conditions, mainly salinity, hindered for centuries the identification of the taxonomic entities encompassed within *Artemia*. In addition, the mismatch between molecular and morphological evolution rates complicates the characterization of evolutionary lineages, generating taxonomic problems. Here, we propose a phylogenetic hypothesis for *Artemia* based on two new complete mitogenomes, and determine levels of congruence in the definition of evolutionary units using nuclear and mtDNA data. We used a fossil of *Artemia* to calibrate the molecular clock and discuss divergence times within the genus. The hypothesis proposed herein suggests a more recent time frame for lineage splitting than previously considered. Phylogeographic analyses were performed using GenBank available mitochondrial and nuclear markers. Evidence of gen e flow, identified through discordances between nuclear and mtDNA markers, was used to reconsider the specific status of some taxa. As a result, we consider *Artemia* to be represented by five evolutionary units: Southern Cone, Mediterranean—South African, New World, Western Asian, and Eastern Asian Lineages. After an exhaustive bibliographical revision, unavailable names for nomenclatural purposes were discarded. The remaining available names have been assigned to their respective evolutionary lineage. The proper names for the evolutionary units in which brine shrimps are structured remain as follows: *Artemia persimilis* Piccinelli & Prosdocimi, 1968 for the Southern Cone Lineage, *Artemia salina* (Linnaeus, 1758) for the Mediterranean-SouthAfrican Lineage, *Artemia urmiana* Günther, 1899 for the Western Asian Lineage, and *Artemia sinica* Cai, 1989 for the Eastern Asian Lineage. The name *Artemia monica* Verrill, 1869 has nomenclatural priority over *A. franciscana* Kellogg, 1906 for naming the New World Lineage. New synonymies are proposed for *A. salina* (*= C. dybowskii* Grochowski, 1896 **n. syn.**, and *A. tunisiana* Bowen & Sterling, 1978 **n. syn.**), *A. monica* (= *A. franciscana* Kellogg, 1906 **n. syn**., and *A. salina* var. *pacifica* Sars, 1904 **n. syn.**); *A. urmiana* (= *B. milhausenii* Fischer de Waldheim, 1834 **n. syn.**, *A. koeppeniana* Fischer, 1851 **n. syn.**, *A. proxima* King, 1855 **n. syn.**, *A. s. var. biloba Entz, 1886* **n. syn.**, *A. s. var. furcata* Entz, 1886 **n. syn.**, *A. asiatica* Walter, 1887 **n. syn.**, *A. parthenogenetica* Bowen & Sterling, 1978 **n. syn.**, *A. ebinurica* Qian & Wang, 1992 **n. syn.**, *A. murae* Naganawa, 2017 **n. syn.**, and *A. frameshifta* Naganawa & Mura, 2017 **n. syn.**). Internal deep nuclear structuring within the *A. monica* and *A. salina* clades, might suggest the existence of additional evolutionary units within these taxa.

## Introduction

Taxonomic practice includes two separated but closely linked activities: the recognition and definition of the biological units resulting from speciation processes and the provision of a universal name for each of those biological units (Wiley, 1981; Minelli, 2003; De Carvalho et al., 2005; Padial et al., 2010). Recognition of biological units follows the classical scientific methodology: observation, hypotheses formulation, data gathering, hypotheses testing, and proposal to the scientific community for further testing, since species are also working hypotheses. Provision of a universal name for each animal is done by strictly applying the rules and recommendations of a code of practice, the International Code of Zoological Nomenclature (International Commission on Zoological Nomenclature, 1999), provided by the International Commission on Zoological Nomenclature.

Historical confusion between these two activities, identification of biological units and naming them, has rendered taxonomy a sort of obscure, almost mystical, discipline, difficult to accommodate to society or even to be understood by non-taxonomist scientists (Rosen, 1986; Dubois, 2003; Lipscomb, Platnick & Wheeler, 2003; Mace, 2004; Wheeler & Valdecasas, 2005; Garnett & Christidis, 2007; Ebach, Valdecasas & Wheeler, 2011).

**Figure 1:**
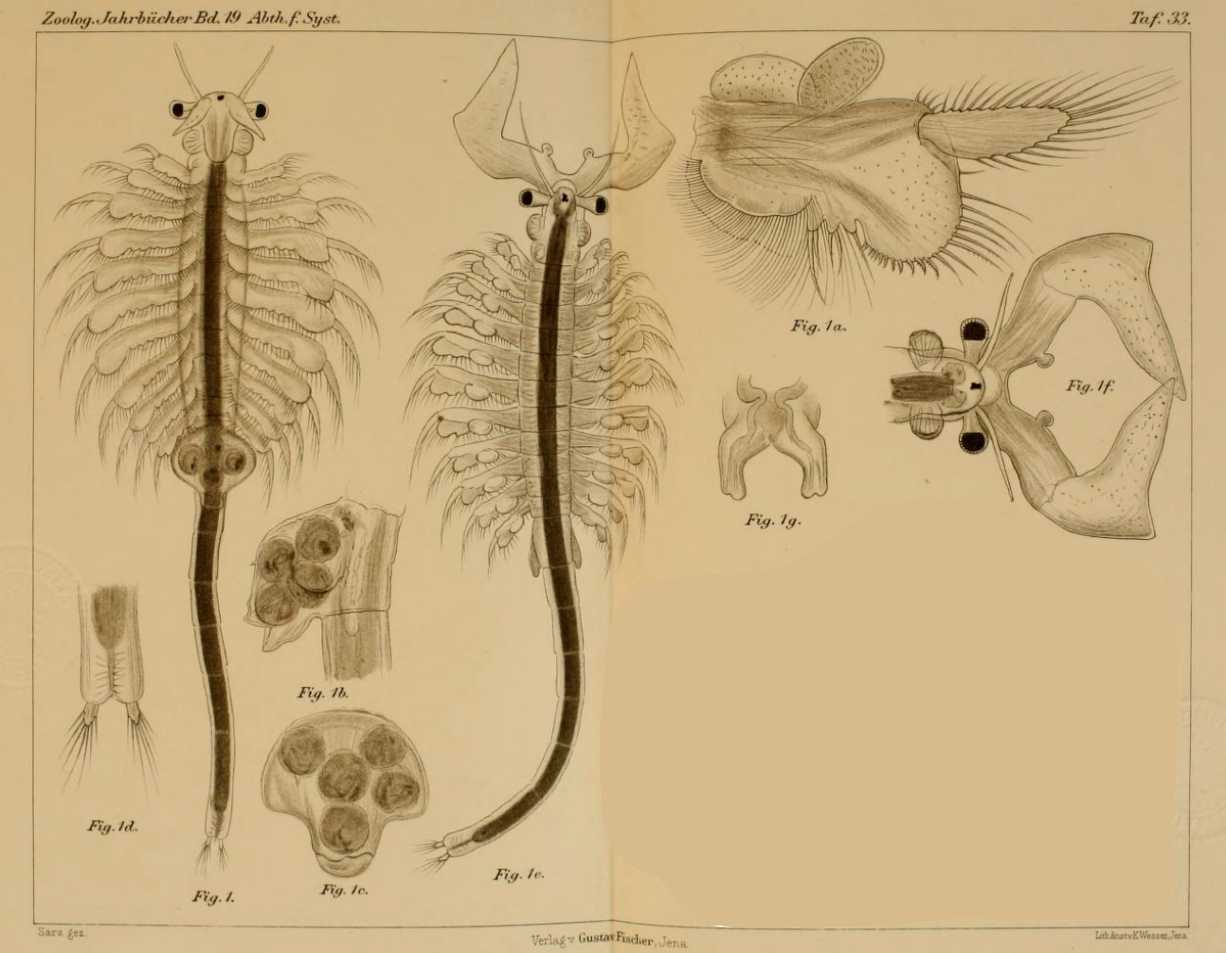
Original illustration of *Artemia salina* var. *pacifica* by Sars (1904) from *Zoologische Jahrbücher*, 19, pl. 33, a high-quality illustration accompanying a precise morphological description of a valid taxon. This is one of the names that, in case molecular data supported their specific ascription, would have nomenclatural priority over *Artemia franciscana* Kellogg, 1906.

The systematics and nomenclature of the brine shrimp (*Artemia* Leach, 1819) is a clear example of the problems that nomenclatural practice, when not carefully considered, can generate when studying model organisms. *Artemia* is a poorly diversified group of small hypersaline water branchiopods (Crustacea, Anostraca), currently conformed by less than a dozen species distributed all over the world, often associated to salt production, and used as a model system for diverse research purposes, as well as a valuable food source in aquaculture (Lenz, 1984; Sorgeloos et al., 1986; Sorgeloos, Dhert & Candreva, 2001; Van Stappen, 1996; Ruebhart, Cock & Shaw, 2008; Amat et al., 2005; Baxevanis, Kappas & Abatzopoulos, 2006). Despite the reduced number of species, the different taxa within *Artemia* have been referred to, in the scientific literature, with more than 50 names, almost all of them used at the species level (Daday de Deés, 1910; Belk & Brtek, 1995; Rogers, 2013; Asem et al., 2020). Most of the names applied from the end of the eighteen to the mid-twentieth century in *Artemia* taxonomic characterization were forgotten and not used again by later authors. Some of those names were not accompanied by adequate descriptions or were applied to populations no longer existing or hard to locate, making difficult their subsequent evaluation and application (Fischer, 1851; King, 1855; Liévin, 1856; Verrill, 1869 in part; Grube, 1874; Walter, 1887; Grochowski, 1896). However, that was not the case for some others (Fischer de Waldheim, 1834; Verrill, 1869 in part; Sars, 1904) (Fig. 1). The abandonment of older names brought a new series of species descriptions, sometimes applied to populations already named, generating nomenclatural problems that required direct actions from the International Commission on Zoological Nomenclature (International Commission on Zoological Nomenclature, 1985, 1993). However, these actions from the ICZN, were not enough to stabilize brine shrimp nomenclature, and still today, some names remain problematic. Reasons for this problematic nomenclatural situation are of diverse nature, some of them intrinsic, directly related to the peculiar biological characteristics of *Artemia*, and some of them extrinsic, related to the human perspective of their study.

Among the intrinsic factors, we may consider first the extreme morphological and physiological phenotypic plasticity shown by *Artemia*. Brine shrimps can change dramatically in size, shape or even degree of development of anatomical structures as a function of the salt concentration at which early stages are exposed during their development (Schmankewitsch, 1875, 1876, 1877a, 1877b; Artom, 1907a, 1907b; Asem & Rastegar-Pouyani, 2010; Asem et al., 2010). A second factor involves the diversity of reproductive modes, ranging from the typical bisexual reproduction in Anostraca, to strict parthenogenesis, and from production of resistance eggs (cysts), through almost ovoviviparity (Artom, 1906a, 1906b, 1906c, 1908; Baxevanis, Kappas & Abatzopoulos, 2006; Maccari et al., 2013, 2014). A third source of conflict is the existence of polyploidy, with 3n, 4n and 5n parthenogenetic specimens that can be found either in syntopy with diploid specimens, or forming populations exclusively conformed by diploid or tetraploid parthenogenetic individuals (Artom, 1913, 1921b; Gross, 1932; Barigozzi, 1934, 1980; Barigozzi & Tosi, 1959; Zhang & Lefcort, 1991; Zhang & King, 1993; Sun et al., 1999; Abatzopoulos et al., 2002b, 2003; Maniatsi et al., 2011; Asem, Eimanifar & Sun, 2016). Although it might seem that reproductive attributes could potentially facilitate the taxonomy of *Artemia*, this diversity was in fact a source of confusion that generated multiple taxonomic descriptions, since names were provided independently for parthenogenetic and bisexual populations.

Taxonomic problems in *Artemia* are related to changes in taxonomic practice over time. The first period of brine shrimp taxonomy was characterized by a proliferation of new species names, defined on the basis of morphological traits later shown to be plastic, and generally applied to populations of a single saltern or salt-lake (Fischer de Waldheim, 1834; Fischer, 1851; Liévin, 1856; Entz, 1886; Verrill, 1869; Grube, 1874; Walter, 1887; Grochowski, 1896; Günther, 1899; Sayce, 1903; Kellogg, 1906). A second historical period involved definition of species based on reproductive mode and laboratory reproductive isolation, coupled or not with protein or cytogenetic analyses. During this period, previously considered units were redefined yielding a new set of names (Piccinelli & Prosdocimi, 1968; Bowen & Sterling, 1978; Barigozzi, 1980; Cai, 1989a, 1989b; Browne & Bowen, 1991; Pilla & Beardmore, 1994). The third and current period of species delimitation, based mainly on molecular DNA information, generated a few more species names and turned species delimitation based almost exclusively on mitochondrial sequence analyses (Asem, Eimanifar & Sun, 2016; Naganawa & Mura, 2017). In addition to all of these numerous taxonomic proposals, it is necessary to remark a poorly done nomenclatural work, sometimes neglecting basic priority principles, ignoring previous species descriptions, or presenting vague type localities, or even not designating type specimens (Kellogg, 1906; Bowen & Sterling, 1978; Abatzopoulos, Zhang & Sorgeloos, 1998). It is difficult to believe that a proper revision of the nomenclature in accordance to the rules and recommendations of the International Code of Zoological Nomenclature (International Commission on Zoological Nomenclature, 1999) has not been performed yet for one of the most world-wide commercialized invertebrates. Only Asem, Rastegar-Pouyani & De Los Ríos-Escalante (2010) made a clarification attempt, and recently, Asem et al. (2020) reviewed the taxonomic problems of native Asian *Artemia*. The task has been probably avoided either because the early inclusion of partial genetic data in the definition of taxa blurred the overall picture (Alonso, 1996), or because the early proliferation of names made the selection of valid names for the molecularly defined taxa a complicated task. Worldwide monographs or catalogues of Anostraca included all names under the synonymy of *Artemia salina* (Linnaeus, 1758) (Linder, 1941; Botnariuc & Orghidan, 1953), or more recently, considered many available names as *nomina nuda* (Belk & Brtek, 1995; Rogers, 2013).

Recently, different research teams have been trying to disentangle the taxonomic problems derived from the complex biology of brine shrimps (Baxevanis, Kappas & Abatzopoulos, 2006; Muñoz et al., 2008; Kappas et al., 2009; Kappas, Baxevanis & Abatzopoulos, 2011; Maniatsi et al., 2011; Maccari, Amat & Gómez, 2013; Maccari et al., 2014; Eimanifar et al., 2014; Asem, Eimanifar & Sun, 2016). These researchers have successfully dealt with the origin and relationships of the parthenogenetic strains, and the evolutionary relationships of the polyploid populations. However, the nomenclatural acts necessary to fix the taxonomic situation of the already identified units cannot be undertaken without a full review of the current set of nomenclatural problems. This situation needs to be sorted out, including the identification of truly problematic areas that have direct consequences on species identification, conservation, or economic impact. In this work, we tried to achieve two goals; first, to present an informed hypothesis on how many singular and evolutionary independent taxa can be defined to date within *Artemia* following the evolutionary species concept (Wiley, 1978), and second, to identify the correct name for each of the biological entities (e.g., species) recovered.

To accomplish this goal, we (1) provide a new mitogenomic robust phylogenetic hypothesis for *Artemia*, with the inclusion of the first mitogenome of the bisexual *A. salina*, and of a Mexican population of *A. franciscana* (= *A. monica*); (2) propose a documented hypothesis on how many evolutionary independent taxonomic units are recognizable within *Artemia* by evaluating levels of congruence between already published mitochondrial DNA (mtDNA) and nuclear data, including fast evolving genes; and (3) identify the biological meaning and identity of each of the published names applied to populations of *Artemia*. In order to accomplish the latter objective, we searched for all the information available in the original bibliographical sources, including original descriptions, reproduction mode, ploidy level, and geographic location of the populations from where names were published.

## Materials and Methods

### Mitogenome analyses

Adult specimens from Laguna Ojo de Liebre, Guerrero Negro, Baja California Sur (BCS) (Mexico) (Arthropod Collection of Museo Nacional de Ciencias Naturales, MNCN 20.04/12541), and of *A. salina* from Salobrar de Campos, Es Trenc, Mallorca (Spain) (MNCN 20.04/12092), stored in absolute ethanol, were used for this study. One specimen of each locality was sent to AllGenetics for DNA extraction and high-throughput sequencing. Briefly, total genomic DNA was extracted using the “RealPure MicroSpin kit” (Durviz®) following the protocol described by the manufacturer. Libraries were prepared using the Nextera DNA Library Prep kit (Illumina, San Diego, CA, USA) and sequenced in an Illumina HiSeq 4000 PE100 lane. Raw data were first cleaned using the R package BBmap (sourceforge.net/projects/bbmap). Genome assembly of the Mexican specimen was carried out using as reference a sequence of the complete *cox1* gene of a record named as *A*. *franciscana* available in Genbank (accession number: NC001620.1), whereas for the sample of *A*. *salina* a partial sequence of *cox1* was used as seed (accession number: KX925417.1), and to avoid possible bias, checked against EU543451.1 (Muñoz et al., 2008). Finally, annotation was performed using the MITOchondrial genome annotation server 2 (MITOS2) (Bernt et al., 2012), checking manually the start and stop codons of all coding genes. The circular map of the *Artemia* mitogenome and its constituent genes are represented in Fig. S1. Mitogenomic annotations are specified in Tables S1 and S2. Newly generated mitogenomes were deposited in Genbank with the accession numbers MT495440 and MT495441, respectively.

We gathered all the complete mitogenomes of Anostraca published in the literature and available in GenBank to construct a data set composed of: a mitogenome of *A. franciscana* from San Francisco Bay (NC001620.1) (Perez et al., 1994; Valverde et al., 1994), a mitogenome of *A. sinica* (MK069595.1) (Asem et al., 2019), another of *A. urmiana*, and two mitogenomes from specimens of two populations of Tibet (identified as *A. tibetiana*, NC021382.1, JQ975177.1, JQ975178.1 respectively) (Zhang et al., 2013). Finally, to include a sample of *A. persimilis* we merged partial mitogenomic sequences of three genes (*cox1*, 12S, and 16S) derived from two different Argentinian samples (KX925418, KX925432 (Qian, Yuangao & Liying, GenBank); FJ007810 (Kappas et al., 2009)). Mitogenomes of *Streptocephalus sirindhornae* Sanoamuang et al., 2000 (KP273593.1 (Liu et al., 2015)) and of *Phallocryptus tserensodnomi* Alonso & Ventura, 2013 (NC026710 (Fan, Lu & Yang, 2016)) were included as outgroups.

For phylogenetic reconstruction purposes, we considered only protein-coding and ribosomal RNA genes, since tRNA genes are highly conserved and resulted to be non-informative. We first extracted a matrix for each protein-coding gene, then we aligned each gene matrix based on their corresponding amino acid translations according to the invertebrate mitochondrial genetic code using the TranslatorX Web Server (Abascal, Zardoya & Telford, 2010) by selecting the MAFFT algorithm (Katoh et al., 2005). We allowed TranslatorX to determine the most likely reading frame. We cleaned the matrixes by removing poorly aligned sites under the Gblocks protein information criterion (Castresana, 2000). For a less stringent selection of the positions to be discarded we allowed gap positions within the final blocks, and for a more stringent selection we did not allow many contiguous non-conserved positions (Abascal, Zardoya & Telford, 2010). RNA genes were aligned and cleaned through the MAFFT and Gblocks online services (Katoh, Rozewicki & Yamada, 2017; Talavera & Castresana, 2007). PartitionFinder v2 (Lanfear et al., 2017) was used to select the best partition scheme and molecular evolutionary models under the Bayesian Information Criterion (BIC; Schwarz, 1978) (Table S2). Because of previous reports of accelerated nucleotide rates (Crease, 1999; Hebert et al., 2002), we tried to reduce the possible effect of saturation by using a data set including only amino acid sequences of coding mtDNA plus ribosomal genes.

Phylogenetic reconstruction was performed using a Bayesian Inference approach implemented in MrBayes version 3.2.6 (Ronquist et al., 2012), using the amino acid + ribosomal concatenated data. MrBayes analyses consisted of two simultaneous runs of 100 million generations each, sampling trees every 10,000 generations. Mixing and convergence among runs were evaluated by checking the average standard deviation of split frequencies, the EES values and the Potential Scale Reduction Factor (PSRF) for each parameter. A majority consensus tree was reconstructed after discarding the first 2,000 sampled trees as burn-in.

**Figure 2:**
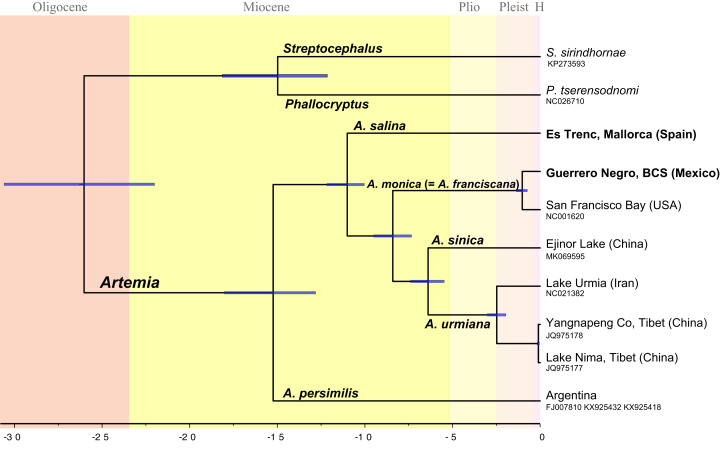
Chronogram showing lineage divergence times in *Artemia* obtained using BEAST following the first scenario hypothesis (Scheme 1). Time indicated in million years (Ma). Dark blue horizontal bars represent 95% HPD (High Posterior Density). A posterior probability value of 1 was obtained for all nodes.

Divergence times across taxa within *Artemia* (Fig. 2) were estimated using Bayesian relaxed molecular clocks implemented in BEAST version 1.8.2 (Drummond et al., 2012). In order to calibrate the molecular clock, we used information derived from fossil specimens originally identified as *A*. *salina* from the Messinian Kalavasos Formation in Cyprus (Manzi et al., 2016). Since the identification of the fossil at the species level is difficult to determine, we considered two alternative scenarios where the fossil might be differently placed. A first scenario (Scheme 1) followed the identification of Manzi et al. (2016) and the fossil was set at the node which clusters all species of *Artemia* excluding *A. persimilis* (Fig. 2). Alternatively, the fossil was treated as a member of the Asian clade (Scheme 2) and thus, the calibration point was settled at the node which clusters the Asian species (Fig. S2). These analyses were performed on the concatenated data set partitioned by gene. This matrix was composed of 13 partitions, the first two corresponding to the ribosomal genes, and the remaining corresponding to the protein-coding genes, except NAD4 and NAD4L, and ATP6 and ATP8, which were merged within the same partition. Site models as well as molecular clocks were unlinked across genes. Trees were linked to ensure that all partitions shared the same tree topology. We used uncorrelated lognormal relaxed clocks with an uninformative prior of substitution rates (gamma distribution, initial value = 0.01, shape = 0.01, offset = 0). Manzi et al. (2016) estimated that the age of the sediments where the fossil was found was about 5.55 Ma. This age was used as a minimum age for the node, a prior with a lognormal distribution (offset = 5.55, mean = 5.55, standard deviation = 0.1), in each of the two proposed scenarios above. Birth-Death model was set as tree prior. The analyses were run for 100 million generations, sampling every 10,000; we inspected the trace plots and effective sample sizes in Tracer 1.8.0 (Drummond & Rambaut, 2007). The first 20 million states were discarded as burn-in. We used Bayes Factor comparison as implemented in BEAST to compare the marginal likelihood value of the two alternative scenarios in which the fossil was placed. To perform marginal likelihood estimations using path sampling (PS)/stepping-stone sampling (Baele et al., 2012) we selected the respective option in the MCMC-BEAUti panel following the default settings. We compared the two marginal likelihood values using the likelihood ratio test, 2Ln (H0–H1). We followed the interpretation of Kass & Raftery (1995) according to which values larger than 2 indicate positive support for one model over the other, and values larger than 6 indicate strong positive support.

Additionally, divergence times across *Artemia* were estimated by using published nucleotide substitution rates to offer a comparison against the results obtained with fossil evidence. In one case, we used the coxI nucleotide substitution rate that was previously used to date phylogenies of Anostraca (Reniers et al., 2013; Lindholm et al., 2016; Rodríguez-Flores et al., 2020) (Scheme 3, Fig. S3). *CoxI* nucleotide substitution rate was estimated from the speciation event of two sister species of snapping shrimps (Decapoda: Alpheidae) separated by the closure of the Isthmus of Panama (Knowlton & Weight, 1998). In a second case, we used the average mitochondrial substitution rate for *Artemia* obtained by Luchetti et al. (2019), who calculated the substitution rates per branch in a time-tree that included representative species of Anostraca, Cladocera and Notostraca (Scheme 4, Fig. S4). For the first case (Scheme 3) we used the concatenated data set partitioned by gene. Site models as well as molecular clocks were unlinked across genes using uncorrelated lognormal relaxed clocks. A lognormal distribution in real space (initial value = 1.0, mean = 0.007, standard deviation = 0.1) was assigned for the ucld.mean parameter of the coxI marker. For the remaining markers, we used uninformative priors (gamma distribution, initial value = 0.01, shape = 0.01, offset = 0). For the second case (Scheme 4), we also used the concatenated dataset with partitions per gene. Site models were unlinked but, differently from the previous case, molecular clocks were linked since the substitution rate was calculated for the entire mitogenome. A lognormal distribution in real space (initial value = 0.0045, mean = 0.0045, standard deviation = 0.18) was assigned for the ucld.mean parameter. The length of the MCMC chain was 100 million generations, sampling every 10,000. Trace plots and effective sample size were inspected in Tracer 1.8.0 (Drummond & Rambaut, 2007). Finally, the first 20 million states were discarded as burn-in.

All analyses were run in the web public resource CIPRES Science Gateway version 3.3 (Miller, Pfeiffer & Schwartz, 2011).

To compare these dating Schemes (1–4) with previous divergence time estimations in *Artemia*, we replicated our analysis using a nucleotide matrix, instead of the amino acid one already used. This matrix was composed of the same 13 partitions explained above. We relaxed the age limits to avoid fixation of narrow bonds imposed by the date of the fossil. Additionally, we replicated the divergence time estimation of Eimanifar, Van Stappen & Wink (2015) using this nucleotide dataset. To accomplish this, we used the estimated age of the node that separates *A*. *salina* from the remaining lineages obtained in Eimanifar, Van Stappen & Wink (2015) (27 Ma, 95% HPD 67.49–10.54 Ma) to calibrate the molecular clock (lognormal distribution, offset = 10.65, mean = 20, standard deviation = 0.6).

### Analyses of available nuclear DNA and mtDNA data

A total of 428 nDNA sequences of the ITS region of *Artemia* (Abatzopoulos et al., 2009; Asem, Eimanifar & Sun, 2016, Asem et al., 2019; Baxevanis, Kappas & Abatzopoulos, 2006; Eimanifar et al., 2014; Kappas et al., 2009; Maccari, Amat & Gómez, 2013; Maniatsi et al., 2009; Valsala, Sugathan & Bharathan, 2015; Vikas et al., 2012) and one of *Streptocephalus proboscideus* (Frauenfeld, 1873) (AY519840) used as outgroup, were downloaded from GenBank and aligned using MAFFT algorithm (Katoh & Toh, 2008). The resulting matrix was cleaned through Gblocks DNA information criterion (Castresana, 2000) excluding several contiguous non-conserved positions and allowing gap positions within the final blocks. A collapsed-haplotype matrix was obtained using the web-based tool ALTER (Glez-Peña et al., 2010) allowing gaps as variable characters. Phylogenetic analyses were performed using a Bayesian Inference approach implemented in MrBayes, using the ITS collapsed data (a total of 226 sequences, including the outgroup). The best substitution model was estimated by setting the command *lset nst* to *mixed*. This procedure results in the Markov chain sampling over the space of all possible reversible substitution models, no matter whether they have a name (e.g., HKY, F81) or not. The analysis consisted of a run of 5 million generations, sampling trees every 1,000 generations.

Because of their different effective population sizes, and being differently conditioned by ploidy and inheritance mechanisms, phylogeographic analyses were performed for nuclear and mitochondrial molecular markers separately (Rodríguez-Flores et al., 2017, 2020). Phylogeographic analyses for the New World Lineage, based on *cox1* mtDNA data, were performed once all sequences from areas falling outside the assumed native distribution of the New World Lineage (American Continent) were removed (Eimanifar et al., 2014; Eimanifar, Van Stappen & Wink, 2015). A *cox1* fragment, extracted from the mitogenome of the specimen from Laguna Ojo de Liebre (Guerrero Negro, Baja California, Mexico) was also included. Sequences were dealt with DNA Sequence Polymorphism version 6.12.01 (Rozas et al., 2017) and collapsed to haplotypes or unique alleles. Gaps in the nuclear marker were treated as variable characters, and consequently a matrix in which each gapped position was considered as a different character was used in the analyses. Networks were constructed through Population Analysis with Reticulate Trees (PopART) 1.7 software (Leigh & Bryant, 2015) applying a TCS algorithm to shape the relationships between alleles. All the information on sequence-haplotype correspondence and their bibliographic sources is shown in Tables 1 and 2.

**Table 1:** Nuclear sequences of the Western Asian Lineage (*A. urmiana*) used in this study.

N° Hap	Sample size	Literature/GenBank referred Taxon*	GenBank accession numbers	Literature source
1	2	A. urmiana*	DQ069926*, DQ084193*	Unpublished data*
2	81	Parthenogenetic; *A. urmiana;*Parthenogens; *Kazakhstan sp., A.urmiana*, Diploid parthenogens, *A. tibetiana;*Diploids, Triploids, Pentaploids; A. parthenogenetica*, A. tibetiana*, *Artemia sp*. Kazakhstan*	DQ201281, DQ201282; MK752753, MK752755, MK752757; FJ004943–FJ004944; KF736247, KF736248, KF736250, KF736253, KF736254, KF736256, KF736260–KF736263, KF736266, KF736267, KF736278, KF736280–KF736285, KF736287, KF736289, KF736290–KF736295; KU183800–KU183804, KU183810–KU183836, KU183843, KU183844, KU183847; MG572086*, MG572087*, MG572089*, MG572092*, MG572093*, MG572099*, MG5720101*, MG5720102*, MG5720104*; DQ069927*; KY000017*, KY000021*	Baxevanis, Kappas & Abatzopoulos (2006), Abatzopoulos et al. (2009), Kappas et al. (2009), Maccari, Amat & Gómez (2013), Asem, Eimanifar & Sun (2016), Unpublished data*
3	4	A. parthenogenetica*	KY000014, KY000015, KY000016*; MG572082*	Unpublished data*
4	2	Diploid parthenogens	KF736274, KF736275	Maccari, Amat & Gómez (2013)
5	1	Diploids	KU183837	Asem, Eimanifar & Sun (2016)
6	2	A. parthenogenetica*	MG572083, MG572091*	Unpublished data*
7	1	*A. sp*. Kazakhstan	DQ084194*	Unpublished data*
8	1	*A. urmiana*	KF736251	Maccari, Amat & Gómez (2013)
9	1	*A. urmiana*	KF736252	Maccari, Amat & Gómez (2013)
10	2	Diploid parthenogens	KF736268, KF736269	Maccari, Amat & Gómez (2013)
11	1	Pentaploids	KU183845	Asem, Eimanifar & Sun (2016)
12	1	Parthenogenetic	DQ201280	Baxevanis, Kappas & Abatzopoulos (2006)
13	5	Tetraploids	KU183805–KU183809	Asem, Eimanifar & Sun (2016)
14	1	A. parthenogenetica*	MG572095*	Unpublished data*
15	5	Tetraploids	KU183838–KU183842	Asem, Eimanifar & Sun (2016)
16	1	Artemia sp.*	DQ069928*	Unpublished data*
17	1	*A. urmiana*	DQ201277	Baxevanis, Kappas & Abatzopoulos (2006)
18	1	Parthenogenetic	DQ201278	Baxevanis, Kappas & Abatzopoulos (2006)
19	1	*A. urmiana*	MK752756	Abatzopoulos et al. (2009)
20	14	Diploid parthenogens; Pentaploids	KF736255, KF736257–KF736259, KF736264, KF736265, KF736270, KF736272, KF736276, KF736277, KF736279, KF736286, KF736288; KU183846	Maccari, Amat & Gómez (2013), Asem, Eimanifar & Sun (2016)
21	2	Diploid parthenogens	KF736271, KF736273	Maccari, Amat & Gómez (2013)
22	1	*A. urmiana*	KF736249	Maccari, Amat & Gómez (2013)
23	1	Parthenogenetic	DQ201279	Baxevanis, Kappas & Abatzopoulos (2006)
24	1	Parthenogenetic	DQ201274	Baxevanis, Kappas & Abatzopoulos (2006)
25	1	Parthenogenetic	DQ201283	Baxevanis, Kappas & Abatzopoulos (2006)
26	1	Artemia sp.*	DQ084195*	Unpublished data*
27	1	A. parthenogenetica*	MG572097*	Unpublished data*
28	1	*A. urmiana*	MK752754	Abatzopoulos et al. (2009)
29	2	A. tibetiana*, A. parthenogenetica*	MG572103*, MG572096*	Unpublished data*
30	2	A. parthenogenetica*	MG572084,85*	Unpublished data*
31	1	A. parthenogenetica*	MG572098*	Unpublished data*
32	10	Eurasian Haplotype Complex (EHC)	KF703803, KF703804, KF703825, KF703830–KF703833, KF703837, KF703841, KF703844, KF703853	Eimanifar et al. (2014)
33	1	Eurasian Haplotype Complex (EHC)	KF703792	Eimanifar et al. (2014)
34	1	Eurasian Haplotype Complex (EHC)	KF703851	Eimanifar et al. (2014)
35	1	Eurasian Haplotype Complex (EHC)	KF703835	Eimanifar et al. (2014)
36	9	Eurasian Haplotype Complex (EHC)	KF703769, KF703774, KF703780, KF703828, KF703831, KF703840, KF703843, KF703845, KF703846	Eimanifar et al. (2014)
37	2	Eurasian Haplotype Complex (EHC)	KF703783, KF703829	Eimanifar et al. (2014)
38	2	Eurasian Haplotype Complex (EHC)	KF703775, KF703782	Eimanifar et al. (2014)
39	1	*A. tibetiana*	KF703785	Eimanifar et al. (2014)
40	1	Eurasian Haplotype Complex (EHC)	KF703772	Eimanifar et al. (2014)
41	1	Eurasian Haplotype Complex (EHC)	KF703768	Eimanifar et al. (2014)
42	1	Eurasian Haplotype Complex (EHC)	KF703805	Eimanifar et al. (2014)
43	1	Parthenogenetic	DQ201284	Baxevanis, Kappas & Abatzopoulos (2006)
44	2	*A. tibetiana*	DQ201269, DQ201270	Baxevanis, Kappas & Abatzopoulos (2006)
45	1	*A. urmiana*	DQ201275	Baxevanis, Kappas & Abatzopoulos (2006)
46	1	*A. urmiana*	DQ201276	Baxevanis, Kappas & Abatzopoulos (2006)
47	1	Eurasian Haplotype Complex (EHC)	KF703809	Eimanifar et al. (2014)
48	1	Parthenogenetic	DQ201273	Baxevanis, Kappas & Abatzopoulos (2006)
49	1	Parthenogenetic	DQ201271	Baxevanis, Kappas & Abatzopoulos (2006)
50	1	Parthenogenetic	DQ201272	Baxevanis, Kappas & Abatzopoulos (2006)
51	1	*A. tibetiana*	KF703798	Eimanifar et al. (2014)
52	1	*A. urmiana*	MK691705	Asem et al. (2019)
53	2	*A. urmiana*	MK691706, MK691763	Asem et al. (2019)
54	1	*A. urmiana*	MK691716	Asem et al. (2019)
55	1	*A. urmiana*	MK691711	Asem et al. (2019)
56	1	*A. urmiana*	MK691713	Asem et al. (2019)
57	2	*A. urmiana*	MK691748, MK691757	Asem et al. (2019)
58	1	*A. urmiana*	MK691741	Asem et al. (2019)
59	11	*A. urmiana*	MK691726, MK691727, MK691732, MK691735, MK691738, MK691744, MK691746, MK691749, MK691752, MK691754, MK691760	Asem et al. (2019)
60	1	*A. urmiana*	MK691734	Asem et al. (2019)
61	1	*A. urmiana*	MK691736	Asem et al. (2019)
62	1	*A. urmiana*	MK691737	Asem et al. (2019)
63	2	*A. urmiana*	MK691756, MK691764	Asem et al. (2019)
64	1	*A. urmiana*	MK691718	Asem et al. (2019)
65	1	*A. urmiana*	MK691712	Asem et al. (2019)
66	1	*A. urmiana*	MK691729	Asem et al. (2019)
67	1	*A. urmiana*	MK691742	Asem et al. (2019)
68	1	*A. urmiana*	MK691724	Asem et al. (2019)
69	1	*A. urmiana*	MK691733	Asem et al. (2019)
70	1	*A. urmiana*	MK691740	Asem et al. (2019)
71	1	*A. urmiana*	MK691750	Asem et al. (2019)
72	1	*A. urmiana*	MK691762	Asem et al. (2019)
73	1	*A. urmiana*	MK691761	Asem et al. (2019)
74	1	*A. urmiana*	MK691714	Asem et al. (2019)
75	2	*A. urmiana*	MK691747, MK691751	Asem et al. (2019)
76	1	*A. urmiana*	MK691759	Asem et al. (2019)
77	1	*A. urmiana*	MK691730	Asem et al. (2019)
78	1	*A. urmiana*	MK691717	Asem et al. (2019)
79	2	*A. urmiana*	MK691728, MK691753	Asem et al. (2019)
80	1	*A. urmiana*	MK691743	Asem et al. (2019)
81	1	*A. urmiana*	MK691709	Asem et al. (2019)
82	1	*A. urmiana*	MK691725	Asem et al. (2019)
83	1	*A. urmiana*	MK691708	Asem et al. (2019)
84	1	*A. urmiana*	MK691721	Asem et al. (2019)
85	1	*A. urmiana*	MK691707	Asem et al. (2019)
86	1	*A. urmiana*	MK691731	Asem et al. (2019)
87	1	*A. urmiana*	MK691745	Asem et al. (2019)
88	1	*A. urmiana*	MK691710	Asem et al. (2019)
89	1	*A. urmiana*	MK691739	Asem et al. (2019)
90	1	*A. urmiana*	MK691755	Asem et al. (2019)
91	1	*A. urmiana*	MK691720	Asem et al. (2019)
92	1	*A. urmiana*	MK691719	Asem et al. (2019)
93	1	*A. urmiana*	MK691723	Asem et al. (2019)
94	1	*A. urmiana*	MK691715	Asem et al. (2019)
95	1	*A. urmiana*	MK691722	Asem et al. (2019)
96	1	*A. urmiana*	MK691758	Asem et al. (2019)
97	1	*A. urmiana*	KF703810	Eimanifar et al. (2014)
98	5	*A. urmiana*	KF703811, KF703813, KF703815, KF703819, KF703822	Eimanifar et al. (2014)
99	1	*A. urmiana*	KF703817	Eimanifar et al. (2014)
100	1	*A. urmiana*	KF703824	Eimanifar et al. (2014)
101	1	*A. urmiana*	KF703821	Eimanifar et al. (2014)
102	2	*A. urmiana*	KF703814, KF703820	Eimanifar et al. (2014)
103	1	*A. urmiana*	KF703818	Eimanifar et al. (2014)
104	1	*A. urmiana*	KF703823	Eimanifar et al. (2014)
105	1	*A. urmiana*	KF703812	Eimanifar et al. (2014)
106	1	*A. urmiana*	KF703816	Eimanifar et al. (2014)

**Note:**

Nuclear sequences of the Western Asian Lineage (*A. urmiana*) used in this study. Names used for populations with available gene sequences are those originally mentioned by their respective authors (Literature referred Taxon). In “GenBank accession number” column, a semi-colon separates sequences by groups according to bibliographic sources, as indicated in “Literature source” column. Symbol “*” indicates that the corresponding sequences were not reported in publications.

**Table 2:** MtDNA sequences of the New World Lineage (*A. monica* = *A*. *franciscana*) used in this study.

N° Hap	Sample size	Literature referred Taxon	GenBank accession numbers	Literature source
1	1	*A. franciscana*	KF662979	Muñoz et al. (2013)
2	2	*A. franciscana*	KF662978	Muñoz et al. (2013)
3	1	*A. franciscana*	KF662984	Muñoz et al. (2013)
4	1	*A. franciscana*	KF662980	Muñoz et al. (2013)
5	1	*A. franciscana*	KF662981	Muñoz et al. (2013)
6	1	*A. franciscana*	KF662983	Muñoz et al. (2013)
7	1	*A. franciscana*	KF662982	Muñoz et al. (2013)
8	1	*A. franciscana*	KF662962	Muñoz et al. (2013)
9	1	*A. franciscana*	KF662963	Muñoz et al. (2013)
10	1	*A. franciscana*	KF662967	Muñoz et al. (2013)
11	1	*A. franciscana*	KF662966	Muñoz et al. (2013)
12	1	*A. franciscana*	KF662965	Muñoz et al. (2013)
13	1	*A. franciscana*	KF662964	Muñoz et al. (2013)
14	8	*A. franciscana*	KF662951, KF663001, DQ401271, DQ401273, DQ401277	Muñoz et al. (2013)
15	1	*A. franciscana*	DQ401276	Tizol-Correa et al. (2009)
16	3	*A. franciscana*	DQ401272, DQ401275, DQ401278	Tizol-Correa et al. (2009)
17	1	*A. franciscana*	DQ401274	Tizol-Correa et al. (2009)
18	1	*A. franciscana*	KF663002	Muñoz et al. (2013)
19	1	*A. franciscana*	KF663021	Muñoz et al. (2013)
20	1	*A. franciscana*	KF663020	Muñoz et al. (2013)
21	1	*A. franciscana*	DQ119645	Hou et al. (2006)
22	4	*A. franciscana*	KF691137–KF691139, KF691141	Eimanifar et al. (2014)
23	1	*A. franciscana*	KF691142	Eimanifar et al. (2014)
24	1	*A. franciscana*	KF691140	Eimanifar et al. (2014)
25	3	*A. franciscana*	KF662985	Muñoz et al. (2013)
26	1	*A. franciscana*	KF662986	Muñoz et al. (2013)
27	1	*A. franciscana*	KF662997	Muñoz et al. (2013)
28	1	*A. franciscana*	KF662987	Muñoz et al. (2013)
29	1	*A. franciscana*	KF662988	Muñoz et al. (2013)
30	1	*A. franciscana*	KF662995	Muñoz et al. (2013)
31	1	*A. franciscana*	KF662994	Muñoz et al. (2013)
32	1	*A. franciscana*	KF662989	Muñoz et al. (2013)
33	1	*A. franciscana*	KF662990	Muñoz et al. (2013)
34	1	*A. franciscana*	KF662991	Muñoz et al. (2013)
35	1	*A. franciscana*	KF662993	Muñoz et al. (2013)
36	1	*A. franciscana*	KF662992	Muñoz et al. (2013)
37	1	*A. franciscana*	KF662996	Muñoz et al. (2013)
38	1	*A. franciscana*	KF662955	Muñoz et al. (2013)
39	3	*A. franciscana*	KF662956, KF662958	Muñoz et al. (2013)
40	2	*A. franciscana*	KF662957	Muñoz et al. (2013)
41	1	*A. franciscana*	KF662961	Muñoz et al. (2013)
42	1	*A. franciscana*	KF663035	Muñoz et al. (2013)
43	4	*A. franciscana*	KF663022, KF663024	Muñoz et al. (2013)
44	1	*A. franciscana*	KF663032	Muñoz et al. (2013)
45	1	*A. franciscana*	KF663031	Muñoz et al. (2013)
46	1	*A. franciscana*	KF663025	Muñoz et al. (2013)
47	2	*A. franciscana*	KF663023	Muñoz et al. (2013)
48	1	*A. franciscana*	KF663034	Muñoz et al. (2013)
49	1	*A. franciscana*	KF663033	Muñoz et al. (2013)
50	1	*A. franciscana*	KF662974	Muñoz et al. (2013)
51	1	*A. franciscana*		This study
52	3	*A. franciscana*	KF691435, KF691437, KF691438	Eimanifar et al. (2014)
53	5	*A. franciscana*	KF663000, KF663003; KF691320, KF663022	Muñoz et al. (2013), Eimanifar et al. (2014)
54	1	*A. franciscana*	KF663005	Muñoz et al. (2013)
55	2	*A. franciscana*	KF662959	Muñoz et al. (2013)
56	3	*A. franciscana*	KF662999; KF691319	Muñoz et al. (2013), Eimanifar et al. (2014)
57	1	*A. franciscana*	KF663004	Muñoz et al. (2013)
58	1	*A. franciscana*	KF662998	Muñoz et al. (2013)
59	7	*A. franciscana*	KF662970, KF662976; AB859231	Muñoz et al. (2013, 2014)
60	1	*A. franciscana*	KF663006	Muñoz et al. (2013)
61	1	*A. franciscana*	KF663008	Muñoz et al. (2013)
62	1	*A. franciscana*	KF663007	Muñoz et al. (2013)
63	2	*A. franciscana*	KF662971; AB859232	Muñoz et al. (2013, 2014)
64	1	A. franciscana*	KF663037	Muñoz et al. (2013)
65	1	A. franciscana*	KF663040	Muñoz et al. (2013)
66	1	A. franciscana*	KF663038	Muñoz et al. (2013)
67	1	A. franciscana*	KF663042	Muñoz et al. (2013)
68	1	A. franciscana*	KF663039	Muñoz et al. (2013)
69	1	A. franciscana*	KF663041	Muñoz et al. (2013)
70	56	*A. franciscana*	KF662968;AB859230; KF691384–KF691390, KF691535, KF691537, KF691538, KF691541, KF691543, KF691544, KF691546; KJ863430, KJ863432–KJ863435, KJ863437, KJ863440–KJ863442, KJ863444–KJ863449, KJ863451–KJ863453, KJ863456–KJ863458, KJ863459, KJ863461, KJ863464, KJ863468–KJ863470, KJ863472, KJ863473, KJ863475–KJ863478, KJ863480, KJ863483, KJ863488, KJ863490	Muñoz et al. (2013, 2014), Eimanifar et al. (2014), Eimanifar, Van Stappen & Wink (2015)
71	1	*A. franciscana*	KJ863465	Eimanifar, Van Stappen & Wink (2015)
72	1	*A. franciscana*	DQ119646	Hou et al. (2006)
73	3	*A. franciscana*	KF662969; KJ863463	Muñoz et al. (2013), Eimanifar, Van Stappen & Wink (2015)
74	2	*A. franciscana*	KF691539; KJ863454	Eimanifar et al. (2014), Eimanifar, Van Stappen & Wink (2015)
75	1	*A. franciscana*	KJ863462	Eimanifar, Van Stappen & Wink (2015)
76	22	*A. franciscana*	KF662977; AB859239; KF691536, KF691540, KF691542, KF691545; KJ863431, KJ863436, KJ863438, KJ863439, KJ863443, KJ863455, KJ863460, KJ863466, KJ863467, KJ863471, KJ863474, KJ863479, KJ863481, KJ863482, KJ863487, KJ863489	Muñoz et al. (2013, 2014), Eimanifar et al. (2014), Eimanifar, Van Stappen & Wink (2015)
77	1	*A. franciscana*	KJ863485	Eimanifar, Van Stappen & Wink (2015)
78	1	*A. franciscana*	KJ863484	Eimanifar, Van Stappen & Wink (2015)
79	1	*A. franciscana*	KJ863486	Eimanifar, Van Stappen & Wink (2015)
80	1	*A. franciscana*	KJ863450	Eimanifar, Van Stappen & Wink (2015)
81	9	*A. franciscana*	KF662960; AB859233	Muñoz et al. (2013, 2014)
82	1	*A. franciscana*	KF662972	Muñoz et al. (2013)
83	1	*A. franciscana*	KF662973	Muñoz et al. (2013)
84	5	*A. franciscana*	DQ401269, DQ401270; KF662975; AB859238	Tizol-Correa et al. (2009), Muñoz et al. (2013, 2014)
85	1	*A. franciscana*	KF691321	Eimanifar et al. (2014)
86	1	*A. franciscana*	KF691323	Eimanifar et al. (2014)
87	3	*A. franciscana*	GU248369–GU248371	Maniatsi et al. (2009)
88	1	*A. franciscana*	GU248372	Maniatsi et al. (2009)
89	2	*A. franciscana*	GU248373, GU248374	Maniatsi et al. (2009)
90	2	*A. franciscana*	GU248379, GU248380	Maniatsi et al. (2009)
91	2	*A. franciscana*	GU248377, GU248378	Maniatsi et al. (2009)
92	2	*A. franciscana*	GU248375, GU248376	Maniatsi et al. (2009)
93	4	*A. franciscana*	KF663009, KF663016	Muñoz et al. (2013)
94	2	*A. franciscana*	KF663010, KF663013	Muñoz et al. (2013)
95	1	*A. franciscana*	KF663012	Muñoz et al. (2013)
96	1	*A. franciscana*	KF663014	Muñoz et al. (2013)
97	3	*A. franciscana*	KF663011, KF663017	Muñoz et al. (2013)
98	1	*A. franciscana*	KF663018	Muñoz et al. (2013)
99	1	*A. franciscana*	KF663019	Muñoz et al. (2013)
100	1	*A. franciscana*	KF663015	Muñoz et al. (2013)
101	1	*A. franciscana*	KF663029	Muñoz et al. (2013)
102	1	*A. franciscana*	KF663028	Muñoz et al. (2013)
103	1	*A. franciscana*	KF662952	Muñoz et al. (2013)
104	2	*A. franciscana*	KF662953; KF691436	Muñoz et al. (2013), Eimanifar et al. (2014)
105	1	*A. franciscana*	KF663030	Muñoz et al. (2013)
106	1	*A. franciscana*	KF663027	Muñoz et al. (2013)
107	1	*A. franciscana*	KF663026	Muñoz et al. (2013)
108	6	*A. franciscana*	GU248363, GU248364, GU248365, GU248366, GU248367, GU248368	Maniatsi et al. (2009)
109	1	*A. franciscana*	GU248362	Maniatsi et al. (2009)

**Note:**

MtDNA sequences of the New World Lineage (*A. monica* = *A*. *franciscana*) used in this study. Names used for populations with available gene sequences are those originally mentioned by their respective authors (Literature referred Taxon). * indicates samples from Mono Lake (California). In “GenBank accession number” column, a semi-colon separates sequences by groups according to bibliographic sources, as indicated in “Literature source” column.

### Nomenclature

An exhaustive bibliographical search was undertaken to locate and gather all original publications in which any possible nomenclatural act affecting *Artemia* was published. The search started with four main sources for synonymies: Daday de Deés (1910), Belk & Brtek (1995), Asem, Rastegar-Pouyani & De Los Ríos-Escalante (2010), Asem et al. (2020), and Rogers (2013). From there, we sought for any additional bibliographic information mentioned in each of the papers consulted. A final search through the Zoological Record database was completed. Each publication was carefully revised in two ways, a first one to obtain data on reproduction mode, ploidy level if available, and precise geographic location of the populations from where names were published; and a second one to evaluate every taxonomic decision made by subsequent authors upon these names in accordance to the rules and recommendations of the International Code of Zoological Nomenclature (International Commission on Zoological Nomenclature, 1999). The second revision included examination of some taxonomic features, including level of detail in the morphological description, designation of type series or holotype, original intention of the author while providing a name (see “Appendix I” for unavailable names), and a subjective evaluation of the methods used to define the evolutionary unit on which the name was applied.

In order to preserve the desired nomenclatural stability, we have tried to assign each of the available names to their respective biological unit. For this task, we used information from type localities (*terrae typicae*) from which taxa were described, because, even if at the time of the description reproductive mode, ploidy, or mtDNA lineage were not recorded, in some cases they were studied subsequently. Problems arose when type locality was not precise, or when introductions were taking place in the area, rendering impossible to determine if the new data gathered actually corresponded to the originally named population (see *nomina dubia* in “Appendix II”). There are names that have been applied historically to parthenogenetic populations, but because they are considered to be the same species as their closely related bisexuals (see below), any of the names applied to parthenogenetic populations are also available for naming the species to which they belong (International Commission on Zoological Nomenclature, 1999).

## Results

### Genome content and organization

The complete mitochondrial genomes of *A. salina* and *A. franciscana* are typical circular DNA molecules of 15,436 bp and 15,825 bp, respectively (Tables S1 and S2; Fig. S1). These mitogenomes encoded the typical 37 genes, including 13 protein-coding genes, 22 transfer RNAs and 2 ribosomal RNAs and a putative mtDNA control region. Like many other mitochondrial genomes of arthropods, the major strand (J strand) carried most of the genes (9 PCGs and 13 tRNAs), while the remaining genes were on the minor strand (N strand). Gene order and orientation were the same as indicated in the previously published *Artemia* mitogenomes (Perez et al., 1994; Valverde et al., 1994; Zhang et al., 2013; Asem et al., 2019).

### Phylogeny of *Artemia*

The topology of the Bayesian phylogram derived from the amino acid + ribosomal concatenated mitochondrial data set was totally congruent with the topology of the ultrametric tree obtained from BEAST (Fig. 2). All nodes are supported with a posterior probability of 1 (PP).

The obtained temporal schemes of diversification in *Artemia* differ markedly depending on the type of evidence used to calibrate the molecular clock (Fig. 2; Figs. S2, S3 and S4) (Table 3). For example, the earliest diversification event within the genus took place in the Late Miocene according to Scheme 3, or in the Paleocene (Scheme 4). The ages of the speciation events within *Artemia* under the different schemes are summarized in Table 3.

**Table 3:** Comparison of different temporal diversification schemes in *Artemia*.

Node	Node description	Scheme 1 Mean/95%HPD Ma*	Scheme 2 Mean/95%HPD Ma	Scheme 3 Mean/95%HPD Ma	Scheme 4 Mean/95%HPD Ma	Scheme 5 Mean/95%HPD Ma
1	First diversification event within *Artemia*	15.29/18.15–12.82	26.34/32.01–21.44	9.73/13.43–6.56	60.55/97.36–36.39	–
2	Split between *A. salina* and the Asian + *A. monica* Clade	11.02/12.19–10.04	19.02/22.12–16.35	7.22/9.89–5.18	47.42/77.04–25.32	27/67.49–10.54
3	Split between *A. monica* and the Asian lineage	8.42/9.55–7.32	14.52/16.55–12.66	5.51/7.59–3.97	36.71/60.93–18.93	34.01/65.42–16.96
4	Speciation event that originated *A. urmiana* and *A. sinica*	6.6/7.40–5.47	11.03/12.15–9.98	3.92/5.37–2.75	24.48/43.15–12.34	19.99/36.69–9.37

**Note:**

Comparison of different temporal diversification schemes in *Artemia*. Scheme 1: using the fossil of *Artemia* described by Manzi et al. (2016) to date the split between *A. salina* and the Asian + *A*. *monica* Clade; Scheme 2: using the *Artemia* fossil described by Manzi et al. (2016) to date the ancestral node of Asian *Artemia*; Scheme 3: using the coxI nucleotide substitution rate estimated for Alpheidae (Decapoda) (Knowlton & Weight, 1998); Scheme 4: using a “total mitogenomic” nucleotide substitution rate for *Artemia* (Luchetti et al., 2019); Scheme 5: using a fossil of *Daphnia* (Eimanifar, Van Stappen & Wink, 2015) (notice that Eimanifar, Van Stappen & Wink, 2015, tree topology differs from ours in the relative position of *A*. *monica* = *A. franciscana* and *A. salina*). *Ma stands for Mega anum (1,000,000 years).

Bayes Factor comparison between the model marginal likelihoods of Schemes 1 and 2, favors scheme 1 hypothesis: 2lnBF = 2*((−34245.58) − (−34274.06)) = 58.96, which, according to the scale given in Kass & Raftery (1995), can be interpreted as very strong support in favor of Scheme 1.

All phylogenetic analyses yielded the same tree topology. This topology is described below, incorporating the TMRCAs corresponding to Scheme 1. The sample representing the Southern Cone lineage (*A. persimilis*) is sister to a clade that includes all the remaining ingroup samples (PP = 1); the splitting event between *A. persimilis* and the ancestor of all the remaining *Artemia* took place about 15.3 Ma (95% HPD 18.15–12.8 Ma). This separation event coincides with the split of the outgroup species (*P. tserensodnomi* and *S. sirindhornae*). A subsequent speciation event, 11.0 Ma (95% HPD 12.19–10.04 Ma), separated the Mediterranean-South African lineage (*A. salina*) from the ancestor of all other taxa during the Late Miocene. The clade composed by the North American samples (*A. monica*, see taxonomic discussion) is sister to the Asian Clade (PP = 1). These two clades started to diverge about 8.4 Ma (95% HPD 9.55–7.32 Ma). The two specimens that conform the North American lineage, Guerrero Negro and San Francisco Bay, diverged in the Pleistocene, 1 Ma (95% HPD 1.37–0.72 Ma). Separation between the Eastern (*A. sinica*) and Western (*A. urmiana*) Asian lineages occurred about 6.6 Ma (95% HPD 7.40–5.47 Ma). Mitogenome information suggests that historical isolation within the Western Asian lineage started 2.4 Ma (95% HPD 3.02–1.95 Ma) by the divergence of Tibetan populations from the remaining populations that conform this clade.

**Figure 3:**
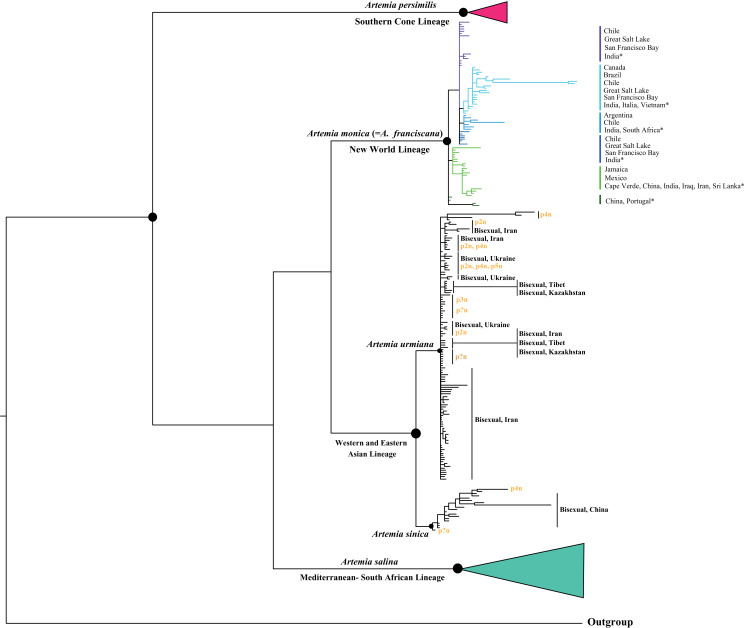
Bayesian phylogenetic relationships of *Artemia* based on nuclear *ITS1* region sequences (see Materials and Methods for sequence original sources). Note the position of populations form Tibet and Kazakhstan. Posterior probabilities >0.90 indicated by black dots.

The Bayesian analysis of the nuclear marker dataset (ITS region) generated a tree constituted by five well-supported clades (Fig. 3). Main clades show posterior probabilities between 0.90 and 1 (black spots), although relationships among them are not always fully resolved: The Southern Cone Lineage constitutes a well-defined clade and includes bisexual populations from Chile (Pichilemu and Torres del Paine), and Argentina (Buenos Aires) (Baxevanis, Kappas & Abatzopoulos, 2006; Kappas et al., 2009). The New World Lineage is conformed by some well differentiated internal clades, in which specimens from populations from Argentina, Brazil, Canada, Chile, Mexico, Jamaica and USA (Great Salt Lake and San Francisco Bay) are included. Introduced populations from Cape Verde, China, India, Iraq, Iran, Italy, Portugal, South Africa, Sri Lanka and Vietnam (Baxevanis, Kappas & Abatzopoulos, 2006; Kappas et al., 2009; Maniatsi et al., 2009; Vikas et al., 2012; Eimanifar et al., 2014; Valsala, Sugathan & Bharathan, 2015), fall also in this clade. The Asian Lineage is formed by two well defined and separated clades: Western and Eastern Asian clades. The Western clade contains bisexual populations from Iran, Ukraine, Tibet and Kazakhstan, and diploid, triploid, tetraploid and pentaploid parthenogenetic populations from Azerbaijan, China (including Tibetan populations), India, Iraq, Iran, Kazakhstan, Pakistan, Russia, Turkey, Turkmenistan, Ukraine, Uzbekistan; in addition, it includes also parthenogenetic populations form Albania, Egypt, Greece, Italy, Israel, Madagascar and Namibia (Baxevanis, Kappas & Abatzopoulos, 2006; Abatzopoulos et al., 2009; Kappas et al., 2009; Maccari, Amat & Gómez, 2013; Eimanifar et al., 2014; Asem, Eimanifar & Sun, 2016; Asem et al., 2019). The relationships among populations within the Western Asian Clade remain unresolved. The Eastern Asian clade includes bisexual and parthenogenetic populations from different Chinese locations (Kappas et al., 2009; Maccari, Amat & Gómez, 2013; Eimanifar et al., 2014). Finally, the Mediterranean—South African clade is formed by bisexual populations from Algeria, Cyprus, Egypt, Italy, Libya, Spain, South Africa and Tunisia (Baxevanis, Kappas & Abatzopoulos, 2006; Eimanifar et al., 2014).

### Phylogeographic analyses

**Figure 4:**
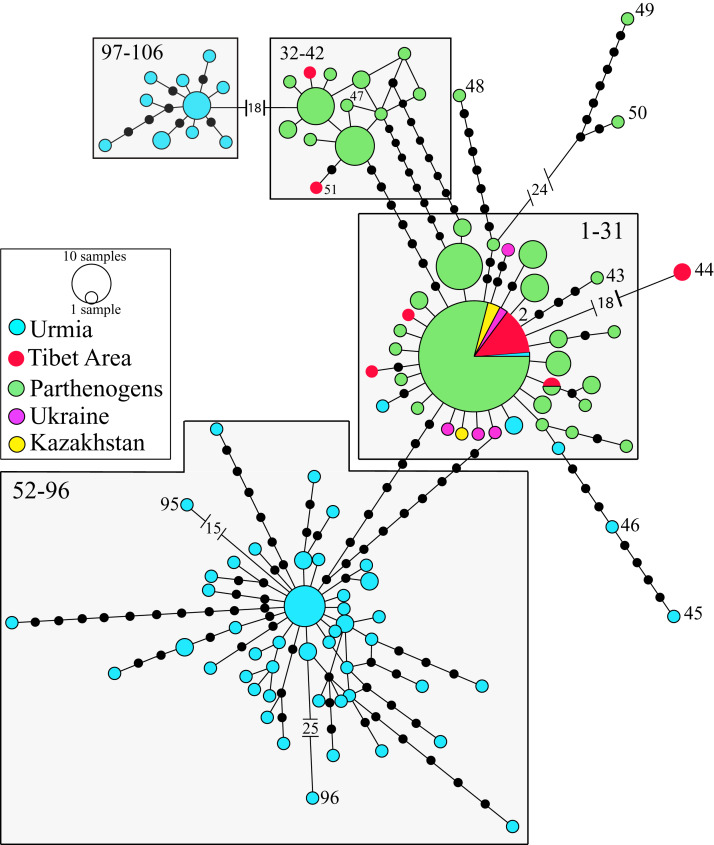
Allelic network of the Western Asian Lineage (*A. urmiana*) based on *ITS1* sequence data (see materials and methods for sequence original sources). Note that most Tibetan specimens (bisexual populations, in red) share a common allele, or differ by a reduced number of nucleotide substitutions with respect to parthenogenetic populations from all over the continent. Haplotype #44 differs from the widespread haplotype #2 in 18 positions affected by a gap, but otherwise it does not show any nucleotidic change. Size of allele circles is proportional to number of individuals. Numbers indicate allele identification. Black dots separating alleles represent individual nucleotide substitutions. A total of 106 alleles were recorded. Information on sequence-allele correspondence is shown in Table 1.

The phylogeographic analysis of the nuclear data set of the Western Asian Clade (*A. urmiana*) includes 106 different nuclear alleles (Fig. 4). Specimens from almost all parthenogenetic populations and the bisexual populations from Tibet, Kazakhstan, Ukraine and Lake Urmia (Iran) all share a common allele (#2). Divergent alleles (#48, 49 and 50) correspond to parthenogenetic individuals from Greece and Israel (Baxevanis, Kappas & Abatzopoulos, 2006) and #95 and #96 to bisexual individuals from Lake Urmia (Asem et al., 2019). Some specimens from Lake Urmia (#97 to #106) (Eimanifar et al., 2014) are genetically distant from all other samples. Tibetan bisexual specimens from LagKor Co (haplotype #44) (Baxevanis, Kappas & Abatzopoulos, 2006) differ from two other bisexual specimens of the same locality (#2, Maccari, Amat & Gómez, 2013) by the presence of a gap involving 18 positions, probably caused by a single evolutionary event, with no additional substitution events occurring between them. Nuclear data do not show geographic structure, including a widely distributed allele #2, suggesting that introgression or gen flow is occurring across Western Asian mtDNA defined clades (Baxevanis, Kappas & Abatzopoulos, 2006; Maniatsi et al., 2011; Asem, Eimanifar & Sun, 2016). In addition, laboratory crosses demonstrated inter-fertility between bisexual populations from diverse Asian localities, from Lake Urmia (Iran) to Catvis (Kazakhstan) (Pilla & Beardmore, 1994).

**Figure 5:**
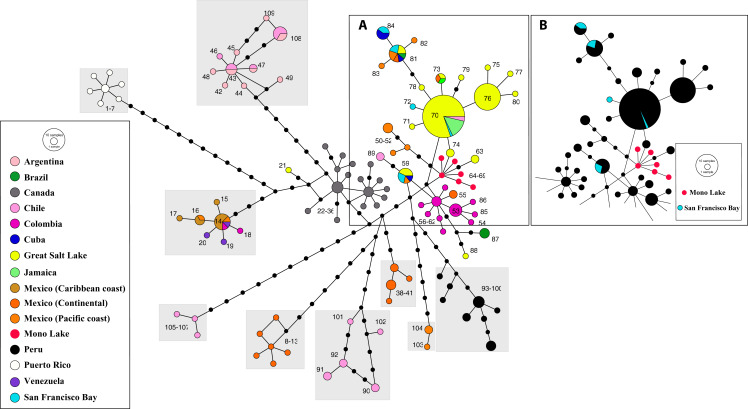
Haplotype network of *cox1* sequence data for the New World Lineage (see Materials and Methods for sequence original sources). (A) Includes most of the available sequences from western US (Great Salt Lake—yellow, San Francisco Bay—light blue, Mono Lake—red), together with clusters of sequences from México (orange) and Colombia (pink), and presumed introduced populations from Jamaica and Cuba. (B) Is identical to (A), with colors changed to remark visually the close allele proximity between Mono Lake (type locality of *A. monica*) and San Francisco Bay (type locality of *A. franciscana*) samples. Nucleotide substitutions between Great Salt Lake and Mono Lake specimens range from 1 to 3. Nucleotide substitutions between Great Salt Lake and San Francisco Bay specimens range from none to 1. According to their position in the network, it is likely that the Great Salt Lake, San Francisco Bay and Mono Lake populations originated from a very recent common ancestor as discussed by Abreu-Grobois & Beardmore (1991). Size of circles proportional to number of individuals sharing haplotype. Numbers identify haplotypes. Black dots separating haplotypes represent individual nucleotide substitutions. A total of 109 haplotypes were included. Information on sequence-haplotype correspondence is shown in Table 2.

Phylogeographic analysis of the New World Clade included 109 different *cox1* haplotypes, published under the names *A. franciscana* and *A. monica* (Fig. 5). The haplotype network displays high geographic structure, including multiple cohesive geographic clusters as those from Peru or Puerto Rico, and divergent populations form Mexico, Chile and Argentina. Haplotypes within the core group (Fig. 5A), including those from the Great Salt Lake, Mono Lake and San Francisco Bay (USA), Mexico (Continental and Pacific Coast), Brazil, Cuba, Colombia, Chile, and Jamaica, differ very little with respect to each other. Haplotypes from Mono Lake (type locality of *A. monica*) are very similar to those from the Great Salt Lake and San Francisco Bay (type locality of *A. franciscana*) (Fig. 5B).

## Discussion

### Phylogeny and time of diversification in *Artemia*

*Artemia* was recovered as a monophyletic lineage in our mitogenomic phylogeny (Fig. 2), with internal phylogenetic relationships clearly depicting a sister taxon relationship between *A. persimilis* and the rest of clades, including Old and New World taxa. Previous authors suggested a similar set of relationships based on nuclear and mitochondrial sequences (Baxevanis, Kappas & Abatzopoulos, 2006; Maniatsi et al., 2011; Eimanifar et al., 2014), enabling the rejection of the reciprocal monophyly of the Old Word vs New World taxa.

The dates for the origin of *Artemia* and of its initial diversification are controversial. Previous authors such as Baxevanis, Kappas & Abatzopoulos (2006) estimated that the origin of *Artemia* occurred 80–90 Ma, whereas Eimanifar, Van Stappen & Wink (2015) proposed a Late Eocene Origin (34.01 Ma, 95% HPD: 16.96–65.42 Ma). Our estimates provide a much more recent date for the origin of *Artemia*. Differences between time estimates presented herein and those proposed in previous studies arise from the type of evidence used to calibrate the molecular clock. Geological information is often used to assign a probable age to nodes affected by certain geological event (Hipsley & Müller, 2014; Ho et al., 2015). However, the assumption of divergence as a consequence of specific geological events represents an independent hypothesis that needs to be properly tested and not merely assumed (Kodandaramaiah, 2011; Magallón, 2004). Baxevanis, Kappas & Abatzopoulos (2006) in a pioneer attempt to date the origin of diversification of *Artemia*, assumed that a series of paleogeographic events were involved in the direct separation of a lineage into a pair of sister taxa, for example, the split of South America from ancient Gondwana in the divergence between the South American *A. persimilis* and the Eurasian lineages. This approach might produce a considerable overestimation of diversification times, aside of underestimating the *cox1* substitution rates in *Artemia* compared to most arthropods (usually ranging from 1.4 to 2.6% per million year) (Knowlton & Weight, 1998). Eimanifar, Van Stappen & Wink (2015), instead, used an indirect approach to calculate divergence times, estimating the separation between Anostraca and Cladocera using as calibration point a fossil of *Daphnia* and including samples of *Artemia* as representatives of Anostraca. However, this approach involved large incomplete sampling, a problem that could affect the estimation of divergence times (Stadler, 2009). Nevertheless, fossils of *Artemia* were unknown by previous authors and divergence times estimated with the indirect approach of Eimanifar, Van Stappen & Wink (2015) provided a novel overview of the evolutionary history of the family.

Records of fossil specimens provide crucial information on the minimum ages of a clade, although its dating and correct phylogenetic placement is sometimes complex (Thorne, Kishino & Painter, 1998; Magallón, 2004). The identification of Manzi et al. (2016) fossils is problematic since the main character which separates *A. salina* from other species of *Artemia* is the absence of a spine at the basis of male penises (Mura & Brecciaroli, 2004), a character that cannot be appreciated in Manzi et al. (2016) fossilized specimens. However, there are some evidences suggesting that the identification of Manzi et al. (2016) fossils as *A. salina* is probably correct. The location of the fossils, Kalavasos Formation in Cyprus (Manzi et al., 2016), practically rules out the possibility that it corresponds to any of the American lineages. In addition, Muñoz et al. (2008) and Baxevanis et al. (2014) demonstrated that *A. salina* shows substantial haplotype diversity that appears geographically structured throughout the Mediterranean. This can be considered as an evidence of the continuous presence of *A. salina* in the Mediterranean area for a very long period of time. Alternatively, the remains could have been part of the Asian Lineage, because they could have been present all over the Eurasian Continent and posteriorly become extinct in the Mediterranean. We have considered this alternative in our dating Scheme 2. The possibility for the fossil to be parthenogenetic, can be ruled out because at least one of the specimens shown by Manzi et al. (2016) is a male. Furthermore, the presumed recent origin of parthenogenesis within Asia (Baxevanis, Kappas & Abatzopoulos, 2006; Maccari, Amat & Gómez, 2013; Maniatsi et al., 2011) would discard such possibility, whereas the fact that the parthenogenetic populations of the Mediterranean share haplotypes with populations from Asia (Maniatsi et al., 2011) is clear signal of their recent arrival to the region.

Considering all available evidences to calibrate the molecular clock and to estimate divergence times within *Artemia*, it seems quite likely that the times of origin and diversification in *Artemia* are much more recent than previously considered (Table 3). Although our estimates might be equally probable than previous hypotheses, we consider our Scheme 1 to be a more realistic scenario (Fig. 2) (Scheme 1 is supported vs Scheme 2 in the Bayes Factor comparisons in BEAST). In addition, the fact that the dates estimated according to scheme 1 are closer to those obtained using the general substitution rate for *cox1* gene (Scheme 3) (Knowlton & Weight, 1998) makes this hypothesis more likely than those requiring substitutions rates much lower than the general rate (Schemes 4 and 5) (Baxevanis, Kappas & Abatzopoulos, 2006; Eimanifar, Van Stappen & Wink, 2015; Luchetti et al., 2019). Luchetti et al. (2019) mutation rate calculated for the entire *Artemia* mitogenome does not consider different molecular clocks for each individual mitochondrial gene. Therefore, until an exhaustive research about specific molecular substitution rates could be carried out for *Artemia*, the tempo of diversification within the genus will remain controversial.

### Evolutionary units within *Artemia* and their nomenclature

Based on the phylogenetic and phylogeographic results presented herein, we consider the genus *Artemia* to be represented by five evolutionary cohesive units (e.g., species), represented by the Southern Cone, Mediterranean—South African, New World, Western Asian, and Eastern Asian Lineages. These units and their nomenclature are discussed in the following paragraphs.

### Southern Cone Lineage—*Artemia persimilis*

The Southern Cone Lineage is a clade geographically restricted to Argentina and Chile. The few populations included in the Southern Cone Lineage are well characterized with respect to the rest of *Artemia* lineages, by morphological, cytogenetic, allozyme, mtDNA, and nuclear sequence features (Halfer-Cervini, Piccinelli & Prosdocimi, 1967; Halfer-Cervini et al., 1968; Piccinelli & Prosdocimi, 1968; Piccinelli, Prosdocimi & Baratelli Zambruni, 1968; Abreu-Grobois, 1987; Badaracco et al., 1987; Hontoria & Amat, 1992a; Amat et al., 1994; Baratelli & Barigozzi, 1990; Gajardo et al., 1995, 2004; Colihueque & Gajardo, 1996; Rodríguez Gil, Papeschi & Cohen, 1998; Cohen et al., 1999; Cohen, Rodríguez Gil & Vélez, 1999; Zúñiga et al., 1999; De Los Ríos & Zúñiga, 2000). This well-defined evolutionary and taxonomic unit, characterized by a particular chromosome number (2*n* = 44; while all other bisexual species present 2*n* = 42) (Abatzopoulos, Kastritsis & Triantaphyllidis, 1986), is sister to all other lineages of *Artemia* (Fig. 2). The Southern Cone Lineage includes geographically structured nuclear (*ITS1*) clades (Baxevanis, Kappas & Abatzopoulos, 2006), congruent with mtDNA data (Gajardo et al., 2004) (Fig. 3).

This clade has been referred to, so far, by a unique species name, *Artemia persimilis* Piccinelli & Prosdocimi, 1968, and except for an unconfirmed report of the species in Italy (Piccinelli & Prosdocimi, 1968; Triantaphyllidis, Abatzopoulos & Sorgeloos, 1998), it has maintained its status as a South American endemic. The synonymic list for *Artemia persimilis* is as follows:

***Artemia persimilis*** Piccinelli & Prosdocimi, 1968

*Artemia persimilis* Piccinelli & Prosdocimi, 1968: 116. Terra typica: “Salinas Grandes di Hidalgo, Argentina”. Holotype and the single paratype indicated, held at Museo Civico di Storia Naturale, Verona, Italy (Piccinelli & Prosdocimi, 1968; Belk & Brtek, 1995).

### Mediterranean-South African Lineage—*Artemia salina*

The Mediterranean-South African Lineage comprises two deep geographically structured mitochondrial clades (South African—Mediterranean), with limited separation between them at the nuclear level (Muñoz et al., 2008; Baxevanis et al., 2014), but markedly divergent at the mtDNA level. Mediterranean populations are on turn structured in a Western and an Eastern main nuclear (*ITS1* and *AFLPs*) clades (Triantaphyllidis et al., 1997a; Baxevanis, Kappas & Abatzopoulos, 2006; Baxevanis et al., 2014). The Mediterranean-South African Lineage includes morphologically and genetically diverse populations, with highly modified local morphotypes, but clearly diagnosable from all other lineages (Amat, 1980a; Triantaphyllidis et al., 1997a; Mura & Brecciaroli, 2004). The Mediterranean-South African Lineage is currently known by the name *A. salina*.

The oldest name for any species of *Artemia*, *Cancer salinus* Linnaeus, 1758, was considered problematic (Bowen & Sterling, 1978). Salt extraction at the type locality of *Cancer salinus* (man-made salterns at Lymington, England) was abandoned, and the brine shrimps disappeared from there, making impossible to collect and study new fresh specimens. We made an inquiry to the Linnean Society (London) to localize any possible material used by Linnaeus (1758) in his description, but the answer was that no material of *Cancer salinus* was currently preserved at the Institution. However, Mura (1990) and Baxevanis, Kappas & Abatzopoulos (2006) located some material from Lymington at the Natural History Museum (London). Their morphological study confirmed that they represent the traditional and current concept of *A. salina* (Mura, 1990; Baxevanis, Kappas & Abatzopoulos, 2006).

Once the name *Artemia salina* (Linnaeus, 1758) is clearly applicable to designate the Mediterranean-South African Lineage (Baxevanis, Kappas & Abatzopoulos, 2006) (neotype designation is however desirable), assignation of additional names to the clade is quite straightforward. Names published for any bisexual taxon in the Mediterranean Region before the introduction of North American specimens a few decades ago, can be undoubtedly assigned to *Artemia salina*. Mura & Nagorskaya (2005) confirmed the presence of *A. salina* as the only bisexual species present at that time in Ukraine, an area of potential contact with bisexual populations of the Western Asian Lineage; this information helped us to retain *A. arietina* Fischer, 1851, under the synonymy of *A. salina*. Two old names with Mediterranean type localities, and whose reproductive mode was not stated in the original description are treated as *nomina dubia*, but tentatively included in the synonymic list for *A. salina*. The resulting synonymic list for the Mediterranean-South African Lineage remains thus as follows:

***Artemia salina*** (Linnaeus, 1758)

*Cancer salinus* Linnaeus, 1758: 634. Terra typica: “Habitat in Angliae Salinis Limingtonianis.”. Bisexual (Linnaeus, 1758). Baxevanis, Kappas & Abatzopoulos (2006) confirmed the morphological ascription of topotypical specimens. Neotype designation among any of those specimens is desirable.

*Gammarus salinus* (Linnaeus, 1758): Fabricius, 1775: 419.

*Artemisia salina* (Linnaeus, 1758): Latreille, 1816: 68. The genus *Artemisia* was supressed for the purposes of the Principle of Priority and placed on the Official Index of Rejected and Invalid Generic Names in Zoology (International Commission on Zoological Nomenclature, 1985, Opinion 1301).

*Eulimene albida* Latreille, 1816: 68 (*nomen dubium*). Terra typica: “…dans la Méditerranée…”. Reproductive mode not indicated (Latreille, 1816). Daday de Deés (1910) included *E. albida* in the synonymy of *A. salina*. The name *Eulimene* Latreille, 1816, does not have nomenclatural precedence over *Artemia* Leach, 1819 (International Commission on Zoological Nomenclature, 1985, Opinion 1301).

*Artemisus salinus* (Linnaeus, 1758): Lamarck, 1818: 135. The genus *Artemisus* was supressed for the purposes of the Principle of Priority (International Commission on Zoological Nomenclature, 1985, Opinion 1301).

*Artemia salina* (Linnaeus, 1758): Leach, 1819: 543.

*Artemia eulimene* Leach, 1819: 543 (*nomen dubium*). Terra typica: “Habite la Méditerranée, près Nice”. Reproductive mode not indicated (Leach, 1819). Desmarest (1823) considered *A. eulimene* a synonym of *Eulimene albida*.

*Artemis salinus* (Linnaeus, 1758): Thompson, 1834: 105.

*Artemia arietina* Fischer, 1851: 156. Terra typica: “… aus der Umgegend von Odessa stammt”. Bisexual (Fischer, 1851). Daday de Deés (1910) included *A. arietina* as a variety of *A. salina*.

*Branchipus (Artemia) salinus* (Linnaeus, 1758): Grube, 1853: 139.

*Branchipus eulimene* (Leach, 1819): Grube, 1853: 140.

*Branchipus arietinus* (Fischer, 1851): Grube, 1853: 140.

*Branchipus oudneyi* Liévin, 1856: 1. Terra typica: “…die Trona-Seen, und besonders der Bahr-el-Dud… Diesen See bewohnt der berühmte Fezzan-Wurm oder Dud” [Lybia: Fezan: Ubari Trona Lake]. Bisexual (Liévin, 1856). Daday de Deés (1910) included *B. oudneyi* in the synonymy of *A. salina*.

*Artemia oudneyi* (Liévin, 1856): Baird in Liévin, 1856: 1.

*Callaonella dybowskii* Grochowski, 1896: 100. **New synonymy**. Terra typica: “im Süsswaser, nämlich im Vrana-See auf der Insel Cherso”. Bisexual populations (Grochowski, 1896). Daday de Deés (1910) commented on the peculiarity of being collected in freshwater at the Croatian Island of Cres (Grochowski, 1896), but treated it as a synonym of *Artemia jelskii* Grube, 1874. According to the illustration provided by Grochowski (1896), it is morphologically assignable to *A. salina*.

*Artemia dybowski* (Grochowski, 1896): Belk & Brtek, 1995: 316.

*Artemia tunisiana* Bowen & Sterling, 1978: 595. **New synonymy**. Terra typica: not stated explicitly, but the authors included two populations in the category: “… from Tunis, and from San Bartolomeo, Sardinia”. Type series or type material not designated. Bisexual populations (Bowen & Sterling, 1978).

### New World Lineage—*Artemia monica* (= *A. franciscana*)

**Figure 6:**
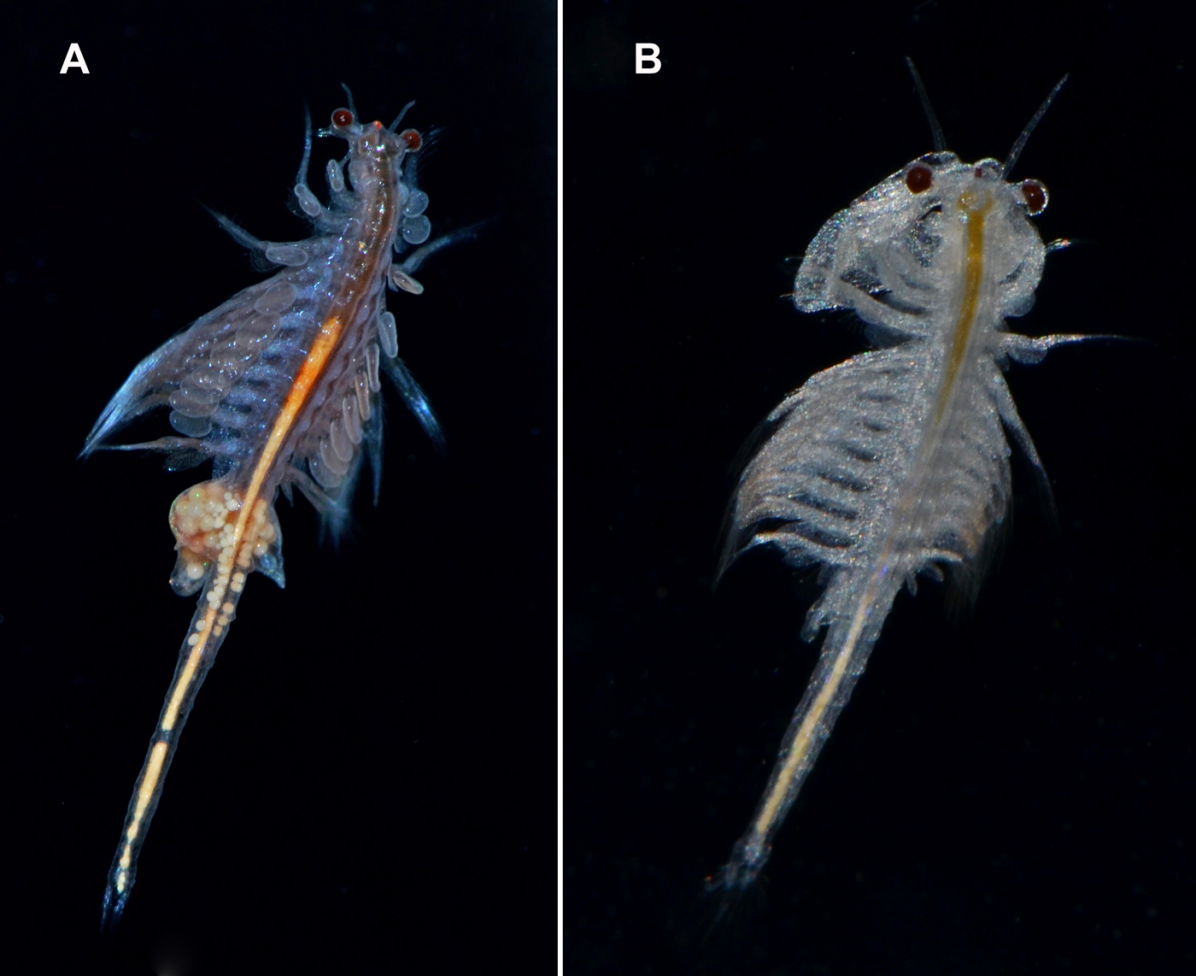
Live specimens of *Artemia monica* (= *A. franciscana*). (A) female; (B) male from an introduced population in Spain (San Fernando, Cádiz). Photographs by M. García-París.

The widely distributed New World Lineage is integrated by multiple geographically structured mitochondrial clades and shows large nuclear sequence variability (Sáez, Escalante & Sastre, 2000; Baxevanis, Kappas & Abatzopoulos, 2006). It has been introduced all over the world (Figs. 6A and 6B) (Eimanifar et al., 2014).

*Cox1* clades within the New World Lineage are largely divergent and, in most cases reciprocally monophyletic and, once excluding the demonstrated introduced populations (Eimanifar et al., 2014), they are geographically structured (Fig. 5). Some of the well-differentiated mtDNA clades are isolated in the Antilles (Puerto Rico), Mexico, or in different areas of South America, and present high intraspecific *F*_ST_ values based on allozyme data (maximum values of *F*_ST_ = 0.24 to 0.38) (Abreu-Grobois & Beardmore, 1980, 1983; Abreu-Grobois, 1983, 1987; Gajardo & Beardmore, 1993; Pilla & Beardmore, 1994). However, there is no evidence supporting that any of these divergent phylogroups might represent a different species. Specimens from divergent mtDNA lineages (all mentioned under the name *A. franciscana*) and different geographic origins occur together in introduced populations through Europe and Asia, providing some indication of the lack of genetic isolation among mtDNA phylogroups (Eimanifar et al., 2014). In addition, Abreu-Grobois & Beardmore (1991) studied 22 allozyme loci and recorded intraspecific *D*_Nei_ distances (0.09–0.13) (Nei, 1972) between populations of the Great Salt Lake (Utah), San Francisco Bay (California), and Pekelmeer (Bonaire). Nevertheless, our mitogenomic data show a relatively large divergence between Baja California (Mexico) and San Francisco Bay (USA) populations (separated about 1,300 km), suggesting that divergent phylogroups within the New World Lineage should be studied at the nuclear level before reaching a final conclusion (as already suggested by Bowen et al., 1985).

The only proposal to consider differentiated taxa within the New World Lineage, was prompted by the ecological isolation of Mono Lake (California) population. Brine shrimps from Mono Lake were considered reproductively isolated from nearby populations because of the particular water ionic composition of the Lake (Clark & Bowen, 1976; Bowen et al., 1985), and accordingly treated as a different species under the name *A. monica* (Conte, Jellison & Starrett, 1988; Dana, Jellison & Melack, 1990, 1995; Dana et al., 1993). The available mtDNA sequences of the endangered *A. monica* are deeply nested within a western USA clade, which includes samples from nearby populations including the Great Salt Lake and San Francisco Bay (type locality of *A. franciscana*), as well as some Mexican and Colombian localities (Figs. 5A and 5B). Samples from Mono Lake do not form a monophyletic mtDNA phylogroup, which probably caused that recent authors totally ignored its existence (Abatzopoulos et al., 2002b; Baxevanis, Kappas & Abatzopoulos, 2006; Maniatsi et al., 2011; Eimanifar et al., 2014). Nuclear data based on 22 allozyme loci do not support the isolation of the Mono Lake population either, since it is deeply nested within a western USA nuclear clade including populations from the Great Salt Lake (Utah), San Francisco Bay (California), and Pekelmeer (Bonaire, Antilles) (Abreu-Grobois & Beardmore, 1991; Pilla & Beardmore, 1994). Nei (1972) genetic distance (*D*_Nei_) between San Francisco Bay (type locality of *A. franciscana*) and Mono Lake (type locality of *A. monica*) populations (*D*_Nei_ = 0.05) falls within the usual range for intraspecific populations, and is even lower than that between San Francisco Bay and the Great Salt Lake populations (*D*_Nei_ = 0.09) (Abreu-Grobois & Beardmore, 1991). In addition, Bowen et al. (1985) demonstrated that in laboratory conditions, under highly controlled chloride and carbonate levels, specimens of *A. monica* (Mono Lake) and of *A. franciscana* from locations nearby show complete reproductive compatibility and a normal mating behavior. All these data already granted rejection of an independent species status for the Mono Lake population by some authors (Abreu-Grobois & Beardmore, 1983; Triantaphyllidis, Abatzopoulos & Sorgeloos, 1998; De Los Ríos & Zúñiga, 2000). In fact, Abreu-Grobois & Beardmore (1991) proposed an appealing explanation for the maintenance of a relatively high level of gene flow between the Mono Lake population and other extant or extinct populations found in the eastern Sierra Nevada mountains. The existence of many saline—carbonated lakes in the Eastern Sierras likely promoted the presence of populations of *Artemia* adapted to local chemical conditions more or less similar to those present today in Mono Lake (Bowen et al., 1985; Abreu-Grobois & Beardmore, 1991). Abreu-Grobois & Beardmore (1991) suggested that climatic and hydrological changes during the Holocene caused sequential extinction and recolonization events as a consequence of variations in the ionic levels of these lakes. Under this circumstances gene flow between Mono Lake and those other populations could have been relatively high, possibly favored by avian movements. This scenario could explain the high allelic diversity found today in Mono Lake and their genetic similarity with respect to other Western US populations (Abreu-Grobois & Beardmore, 1991).

Lack of reciprocal monophyly at nuclear and mtDNA levels suggests that gene flow is effectively taking place between the Mono Lake population and neighboring ones (Fig. 5B). Consequently, the ecological differences observed between the population of Mono Lake and other locations in California and Utah (Lenz, 1980, 1984; Dana & Lenz, 1986), suggest that these populations should be considered either as local adaptive ecotypes (Abreu-Grobois, 1983, 1987), as it is the case for extremely adapted populations in other regions (Schmankewitsch, 1875, 1877a, 1877b; Amat, 1980b; Asem & Rastegar-Pouyani, 2010), or the result of an inconclusive speciation process. Consequently, the Mono Lake brine shrimp together with all western North American *Artemia* are part of a single evolutionary unit.

Belk & Bowen (1990) submitted an application for the conservation of the specific name *Artemia franciscana* Kellogg, 1906, over some of previously published names with nomenclatural priority over it. The Opinion 1704 of the Commission (International Commission on Zoological Nomenclature, 1993) included the names *Artemis guildingi* Thompson, 1834, *Artemia fertilis* Verrill, 1870, and *Artemia utahensis* Lockington, 1876, in the Official Index of Rejected and Invalid Specific Names in Zoology, and gave precedence to *A. franciscana* over *A. gracilis*. However, as a member of the Commission (L.B. Holthius) pointed out, it was premature to deal with the issue before the systematics of *Artemia* was properly analyzed (International Commission on Zoological Nomenclature, 1993). In fact, populations of Mono Lake (type locality of *A. monica*) (Verrill, 1869), and from coastal Peru (where the type locality of *A. jelskii* is located) (Grube, 1874), are likely part of the same taxonomic unit as the populations from San Francisco Bay (type locality of *A. franciscana*), and both have nomenclatural priority over *A. franciscana*. In addition, the detailed description of Hawaiian populations (*A. salina* var. *pacifica*) (Fig. 1; Sars, 1904) also corresponds to the typical morphology of the New World Lineage of *Artemia*, and therefore might have also priority over *A. franciscana*.

The resulting situation is that, besides the Commission’s locked name *A. gracilis* (a *nomen dubium*), at least three other names could have precedence over *A. franciscana* according to the Principle of Priority (*A. monica*, *A. jelskii* and *A. salina* var. *pacifica*). The Code of Nomenclature indicates that the Principle of Priority may be modified in its operation in the interest of stability and universality. The Code estates that: “*23.9.1. prevailing usage must be maintained when the following conditions are both met: 23.9.1.1. the senior synonym or homonym has not been used as a valid name after 1899, and 23.9.1.2. the junior synonym or homonym has been used for a particular taxon, as its presumed valid name, in at least 25 works, published by at least 10 authors in the immediately preceding 50 years and encompassing a span of not less than 10 years*”. The first condition is not met by either Verrill’s (1869) *A. monica* (often used as a valid name after 1899), Grube’s (1874) *A. jelskii* (a *nomen dubium*, but used as a valid species name at least by Daday de Deés (1910) as *Callaonella jelskii*), or Sars’ (1904) *A. salina* var. *pacifica* (described after 1899). The oldest name, *Artemia monica* Verrill, 1869 (published in the same work as *A. gracilis* and therefore without precedence of one over the other), has been used extensively as a valid species name in the XXth and XXIst centuries (see for example Abreu-Grobois & Beardmore, 1991; Brendonck & Belk, 1997; Asem, Eimanifar & Sun, 2016).

The precedence of *A. monica* over *A. franciscana* cannot be reverted under any provision of the Code (International Commission on Zoological Nomenclature, 1999), and thus, *Artemia monica* Verrill, 1869, becomes the valid name for the New World Lineage of Artemia. Therefore, all populations currently referred to by the name *A. franciscana* must be referred to as *A. monica*.

The synonymic list (synonyms and new combinations) for the New World Lineage would remain as follows:

***Artemia monica*** Verrill, 1869

*Artemis guildingi* Thompson, 1834: plate 1, fig. 11 (*unavailable name*). Terra typica: “West Indies”. *Artemis guildingi* Thompson, 1834 was placed in the Official Index of Rejected and Invalid Specific Names in Zoology (International Commission on Zoological Nomenclature, 1993, Opinion 1704).

*? Artemia gracilis* Verrill, 1869: 248 (nomen dubium). Terra typica: “Near New Haven, in tubs of water from salt marsh”. Verrill (1870) precised: “New Haven, Conn. Charlestown, Mass., on railroad bridge across Charles River in tubs of concentrated sea-water.”. Syntypes (396, 397) at the Peabody Museum of Natural History (New Haven, Connecticut, USA) (Belk & Brtek, 1995).

*Artemia monica* Verrill, 1869: 249. Terra typica: Not indicated explicitly in the original description, but a few pages earlier, Verrill (1869: 245) stated “… a number of specimens of a new species, *A. monica*, V., which he collected in Mono Lake, California…”. Syntypes (395) at Peabody Museum of Natural History (New Haven, Connecticut, USA) (Belk & Brtek, 1995). Bisexual (Verrill, 1869).

*Artemia fertilis* Verrill, 1869: 238 (*unavailable name*). Terra typica: “Great Salt Lake, Utah,…”. *Artemia fertilis* Verrill, 1869, was placed in the Official Index of Rejected and Invalid Specific Names in Zoology (International Commission on Zoological Nomenclature, 1993, Opinion 1704).

*? Artemia jelskii* Grube, 1874: 56 (*nomen dubium*). Terra typica: “…Callao…”. Types not designated. Bisexual (Grube, 1874). Daday de Deés (1910) treated it as an independent species as *Artemia* (*Callaonella*) *jelskii* Grube, from salterns near Callao, in Perú. The molecular identification of this population is desirable in order to determine its taxonomic placement and make effective this possible synonymy.

*Artemia utahensis* Lockington, 1876: 137 (*unavailable name*). Terra typica: “… inhabits the Great Salt Lake of Utah.”. *Artemia utahensis* Lockington, 1876, was placed in the Official Index of Rejected and Invalid Specific Names in Zoology (International Commission on Zoological Nomenclature, 1993, Opinion 1704).

*? Callaonella jelskii* (Grube, 1874): Kulczycki, 1885: 591.

*Artemia salina* var. *pacifica* Sars, 1904: 630. **New synonymy**. Terra typica: “… (1) in einem Salzsee, mit 15% Kochsalz, in der Nähe von Honolulu, Hawaiische Inseln, und (2) in einer Lagune, mit 12% Kochsalz, auf der kleinen unbewohnten Koralleninsel Laysan, ungefähr 800 Seemeilen WNW. von Honolulu.”. Bisexual (Sars, 1904; Fig. 1). Although its morphology has been well studied and documented, a molecular identification of this population is desirable.

*Artemia franciscana* Kellogg, 1906: 596. New synonymy. Terra typica: “… found abundantly in the salterns (evaporating pools), density 1.08 to 1.24, at Redwood City, San Francisco Bay,…”. Types not designated (Belk & Brtek, 1995). Bisexual (Kellogg, 1906).

### Western Asian Lineage—*Artemia urmiana*

The Western Asian Lineage is composed of at least three geographically structured mitochondrial clades, some of them including bisexual and parthenogenetic populations (e.g., Urmia Lake), with apparent gene flow among bisexual populations from all three clades (Maccari, Amat & Gómez, 2013; Asem, Eimanifar & Sun, 2016). Bisexual populations are morphologically diagnosable from all other lineages and they have been introduced in diverse areas, including the Mediterranean region.

Mitochondrial clades within the Western Asian Lineage, usually known as *A. tibetiana*, *A. urmiana*, Eurasian Haplotype Complex and *A. parthenogenetica* (in part), are part of a single nuclear clade (Fig. 4) with alleles widely shared across all mtDNA units (Baxevanis, Kappas & Abatzopoulos, 2006; Eimanifar et al., 2014; Asem, Eimanifar & Sun, 2016). Some of these mtDNA clades may represent incipient evolutionary units, with relatively low gene flow occurring among them (Kappas, Baxevanis & Abatzopoulos, 2011; Eimanifar et al., 2014) (Fig. 4). However, Zhang et al. (2013) discussed the low divergence found among complete mitogenomes of bisexual populations from Lake Urmia and Tibet, as reported in previous studies (Baxevanis et al., 2005; Baxevanis, Kappas & Abatzopoulos, 2006; Wang et al., 2008).

Mitochondrial clades may, or may not, represent evolutionary units. If time is long enough, coalescence processes, including lineage sorting, generally will end up by depicting concordant clades for mtDNA and nuclear markers (reciprocally monophyletic if gene flow got interrupted, or single clades if gene flow ended up homogenizing the original incipient clades) (Fujita et al., 2012; Sukumaran & Knowles, 2017; but see Albert, Zardoya & García-París, 2007). However, in many groups, particularly in those with little developed prezygotic isolation mechanisms, mtDNA is often well structured across populations that are still linked by gene flow (García-París et al., 2003; Recuero & García-París, 2011). In these cases, and as a consequence of demographic processes such as maternal inheritance, small population or sampling size, lack of recombination, difficulties to move across contact zones, etc… mtDNA clades can appear as reciprocally monophyletic, transmitting the idea that there has been a long period of isolation between populations, whereas analyses of rapidly evolving nuclear markers such as ITS, show evident signs of gene flow across mtDNA breaks (Babik et al., 2005; Rodríguez-Flores et al., 2017). In these cases, discordances between nuclear and mtDNA markers are very useful to determine isolation levels and consequently the evolutionary status of two population groups. In *Artemia*, which does not show any sign of occurrence of pre-zygotic isolation mechanisms (Pilla & Beardmore, 1994), mitochondrial data have been extensively used to characterize evolutionary units within the Asian Lineages (Eimanifar et al., 2014), or even to describe new taxa (Naganawa & Mura, 2017). However, data from fast evolving nuclear data, mostly ITS1, do not support the recognition of some of those phylogroups or mtDNA clades as independent taxa.

The bisexual population of Lagkor Co in Tibet has been formerly treated as a different species, *A. tibetiana* (Abatzopoulos, Zhang & Sorgeloos, 1998). The taxon was characterized by having cysts with large diameters (323 µm + 17.2; 330 µm +14.6), the longest known first instar nauplii (667 µm + 32.7), and a large adult size (Abatzopoulos, Zhang & Sorgeloos, 1998). *Cox1* mtDNA sequences of *A. tibetiana* clustered in two non-sister clades, sequentially sister to a clade conformed by populations of *A. urmiana* plus 2n and 3n parthenogenetic specimens, rendering *A. tibetiana* a non-monophyletic mtDNA entity (Eimanifar et al., 2014; Asem, Eimanifar & Sun, 2016). Genetic divergence based on allozyme analyses and reproductive incompatibility (postzygotic isolation) between the Tibetan and other Asian populations was relatively low (allozymes), or not significantly different (fertility) from that recorded for intraspecific crossings (40–60% fertile specimens according to Abatzopoulos, Zhang & Sorgeloos, 1998). Abatzopoulos et al. (2002b) pointed out that “The likelihood of extensive geographical differentiation cannot be completely ruled out, especially with the limited number of populations investigated here, a fact that can lead to a fallible taxonomy.” Eimanifar et al. (2014) and Asem, Eimanifar & Sun (2016) phylogeographic analyses based on ITS1 sequence data, which included specimens of all Asian taxa, indicated that the nuclear sequences of the ITS1 region from the type locality of *A. tibetiana*, were almost identical to those of 2n and 3n parthenogenetic specimens and to those of bisexual *A. urmiana* (Fig. 4). Eimanifar et al. (2014) pointed out that: “The presence of a common haplotype can be simply explained because of the lack of time to generate and sort out new variants among closely related species”, while Kappas, Baxevanis & Abatzopoulos (2011) suggested that the large morphological diversity displayed by *A. tibetiana*, coupled with a low level of genetic divergence between *A. tibetiana* and *A. urmiana*, reflects recent speciation or slow rates of divergence. There are thus several evidences against maintaining the species status of the population named *A. tibetiana* from an evolutionary species concept perspective (Wiley, 1978): firstly, the absence of a common ancestor for all populations currently included under this name, that is, the different Tibetan populations do not form a monophyletic group, nor are they evolutionarily cohesive (Maccari, Amat & Gómez, 2013; Eimanifar et al., 2014); secondly, they present unclear boundaries in their genetic/morphological differentiation (Kappas, Baxevanis & Abatzopoulos, 2011), especially considering that gene flow has been occurring across Tibetan and non-Tibetan populations until very recently, as inferred from a rapid evolving nuclear marker (Baxevanis, Kappas & Abatzopoulos, 2006; Maccari, Amat & Gómez, 2013) (Fig. 4). Even though the occurrence of partial cross-fertility in F2 and F3 generations cannot be ignored (Triantaphyllidis, Abatzopoulos & Sorgeloos, 1998; Abatzopoulos et al., 2002b), testing the species hypothesis on the basis of this parameter would require analyzing the reproductive compatibility among other populations of the Western Asian Lineage. We agree with Asem et al. (2020) in that the taxonomic status of some Tibetan populations is dubious until more work on their degree of isolation and population speciation trends is performed. However, these considerations do not affect the status of the name *A. tibetiana* that should be considered as a junior synonym of *A. urmiana* based on the occurrence of nuclear gene flow between the type locality of *A. tibetiana* and populations of *A. urmiana* (Maccari, Amat & Gómez, 2013).

Naganawa & Mura (2017) described recently two Asian *Artemia* species, *A. frameshifta* and *A. murae*. The *cox1* fragment used by Naganawa & Mura (2017) to identify the only female studied *A. frameshifta* (GenBank accession number LC195588) present 11 indels (one to three base pairs long) when aligned with all other sequences of Asian *Artemia*. Amino acid transcription reveals extensive presence of stop codon positions (TAA and TAG) (Eimanifar, 2014) along the first half of the sequence (for all three possible reading frames). Based on the lack of morphological differentiation of *A. frameshifta* and on the affinity of the studied sequence (possibly a pseudogene) with sequences of the Western Asian Lineage, we propose the synonymy of *A. frameshifta* with *A. urmiana*. With regard to *A. murae*, Naganawa provided a well-illustrated morphological description and a *cox1* sequence fragment (GenBank accession number LC195587). A re-examination of this sequence reveals that, a single position base (Adenine) was introduced at the end of the fragment, generating a displacement of the reading frame involving 28 positions. Its amino acid transcription reveals that this sequence presents a large amount of amino acid changes with respect to other Asian *Artemia*, which otherwise present highly conservative amino acid sequences. This fact, together with absence of morphological conclusive differences with respect to *A. urmiana* (as already suggested by Naganawa & Mura, 2017), made us, regretfully, to reconsider the validity of *A. murae* and include it tentatively in the Western Asian Lineage as a junior synonym of *A. urmiana*.

A large number of parthenogenetic populations from the Western and the Eastern Asian Lineages studied shared a general common allele of *Na*^*+*^*/K*^*+*^ *ATPase* (Asem, Eimanifar & Sun, 2016). These data are partially supported by *ITS* data. However, the diversification described for *ITS* is very high (Eimanifar et al., 2014; Asem, Eimanifar & Sun, 2016; Fig. 2), and inconsistent with general patterns of evolution of *ITS* markers in Anostraca (Rodríguez-Flores et al., 2017, 2020). These discordances between sets of markers across Asian populations are not reflected at the morphological level, since bisexual populations from Western and Eastern Asian Lineages seem to differ consistently (*A. urmiana* vs. *A. sinica*) in agreement with mtDNA clades (Cai, 1989b). While these discordances need to be studied at a deeper level, we prefer to retain as separate evolutionary entities the morphologically (nuclear) and mitochondrially defined Western and Eastern Asian Lineages, in agreement with Cai (1989a, 1989b) and Zheng, Sun & Ma (2004) tests of reproductive incompatibility.

Analyses carried out using mtDNA data (*cox1*, *12S* and *16S*), show that all 2n and 3n parthenogenetic specimens cluster together or are nested within a single lineage that also includes some bisexual populations from Lake Urmia, Ukraine, and Tibet (Maniatsi et al., 2011; Asem, Eimanifar & Sun, 2016); mitochondrial differentiation (*cox1*, *12S* and *16S*) between 2n and 3n parthenogenetic populations and bisexual populations from Lake Urmia is quite limited (Baxevanis, Kappas & Abatzopoulos, 2006; Maniatsi et al., 2011; Eimanifar et al., 2014; Asem, Eimanifar & Sun, 2016), suggesting a very recent origin for both parthenogenesis and polyploidy within this clade.

Nuclear data (Fig. 3) suggest that 3n polyploid specimens are occasional evolutionary experiments, likely advocated to extinction, and therefore difficult to be considered as a differentiated taxon (Baxevanis, Kappas & Abatzopoulos, 2006). On the other hand, diploid parthenogenetic populations are well established, and widely distributed, but they will end up as clonal isolated lines, altogether difficult to be considered as a single evolutionary unit or a single taxonomic entity (each female clone is an independent line) (Abatzopoulos et al., 2002a). To complicate matters, the occasional males produced in 2n parthenogenetic populations (MacDonald & Browne, 1987; Mura & Nagorskaya, 2005; Maccari et al., 2013) open a window for the existence of gene flow between males of parthenogenetic origin and bisexual populations when contact is established, which is a relatively frequent situation. This problem requires further analyses, because Baxevanis, Kappas & Abatzopoulos (2006) and Maniatsi et al. (2011) recovered multiple independent origins for parthenogenesis and found large discordances between the evolutionary patterns shown by nuclear and mitochondrial data among parthenogenetic lines. We concur with Baxevanis, Kappas & Abatzopoulos (2006) on considering that 2n and 3n parthenogenetic populations are part of a single Western Asian Lineage and that, at least for the time being, they should not be treated as independent taxa (Fig. 3).

The fact that bisexual, diploid and triploid parthenogenetic populations, are part of a single lineage, highly complicates the nomenclature of the Western Asian Lineage (Asem et al., 2020). There is no information on the level of ploidy or mtDNA data for many of the parthenogenetic populations for which available names have been published, and therefore it is impossible to ascribe those names with certainty to either the Western or the Eastern Asian Lineages. Even having the opportunity to perform molecular analyses of specimens from those localities, the chance that new introductions occurred, would mask original identifications, since 4n (Eastern Asian Lineage) and 2n (Western Asian Lineage) parthenogenetic specimens are currently found together in many areas (Eimanifar et al., 2014). In this sense, some names applied to parthenogenetic populations must remain as *nomina dubia* until additional information can be obtained. Among these are the two Australian taxa described by Sayce (1903), *A. australis* and *A. westraliensis*. Reasons to include them within the Western Asian Lineage are that all parthenogenetic populations so far studied in Australia are reported to be 2n (McMaster et al., 2007; Muñoz & Pacios, 2010).

**Figure 7:**
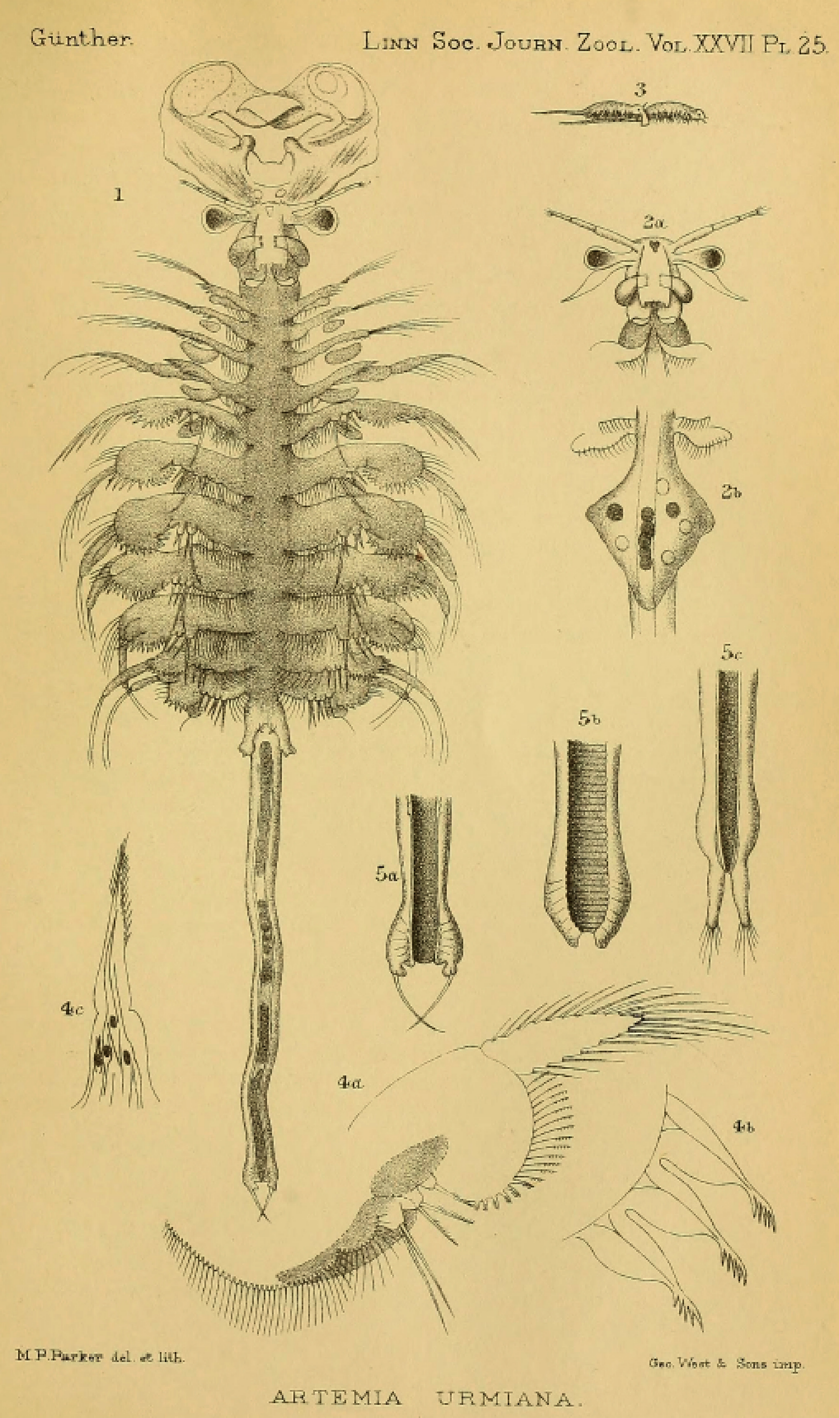
Original illustration of *Artemia urmiana* (*nomen protectum*) in Günther (1899) from *The Journal of the Linnean Society*, 27, pl. 25, a high-quality illustration accompanying the original description of *A. urmiana*.

In order to assure stability, reversion of precedence with respect to *A. urmiana* Günther, 1899, of names applied to parthenogenetic populations described before 1899 (*Branchipus milhausenii* Fischer de Waldheim, 1834; *Artemia koeppeniana* Fischer, 1851, *Artemia proxima* King, 1855, *Artemia salina* var. *biloba* Entz, 1886, *Artemia salina var. furcata* Entz, 1886, and *Artemia asiatica* Walter, 1887) is feasible, because as far as we have been able to find, none of them was used as a valid taxon name after 1899 (International Commission on Zoological Nomenclature, 1999). In fact, Daday de Deés (1910) included all of them, except *A. asiatica* (probably a *lapsus*, because Daday de Dées mentioned the type locality of *A. asiatica* as part of the *A. salina* geographic range), as intraspecific variants or synonyms of *A. salina*, and were not used again as names for any valid taxon. On the other hand, the name *A. urmiana* has been used extensively, in at least 25 works, published by at least 10 authors in the immediately preceding 50 years and encompassing a span of not less than 10 years (see for example: Barigozzi et al., 1988; Abreu-Grobois & Beardmore, 1991; Browne & Bowen, 1991; Triantaphyllidis, Abatzopoulos & Sorgeloos, 1998; Abatzopoulos et al., 2006, 2009; Baxevanis, Kappas & Abatzopoulos, 2006; Eimanifar, Rezvani & Carapetian, 2006; Eimanifar et al., 2014, 2016; Asem & Rastegar-Pouyani, 2007, 2008, 2010; Asem, Rastegar-Pouyani & Agh, 2007; Asem, Rastegar-Pouyani & De Los Ríos-Escalante, 2010; Asem et al., 2010; Asem, 2008; De Los Ríos & Asem, 2008; Shadrin et al., 2008; Agh et al., 2009; Khomenko & Shadrin, 2009; Ahmadi et al., 2012; Anufriieva & Shadrin, 2012, 2013; Castro Mejía et al., 2013; Eimanifar & Wink, 2013; Zhang et al., 2013; Asem, Eimanifar & Sun, 2016; Naganawa & Mura, 2017; and additional references in Asem & Rogers, 2012). Therefore, and according to the Article 23.9.2. of the International Code of Zoological Nomenclature (International Commission on Zoological Nomenclature, 1999), the name *A. urmiana* Günther, 1899 can be considered a *nomen protectum* having thus nomenclatural precedence over the names *Branchipus milhausenii* Fischer de Waldheim, 1834, *Artemia koeppeniana* Fischer, 1851, *Artemia proxima* King, 1855, and *Artemia asiatica* Walter, 1887 (all of them *nomina oblita*). Günther (1899) description of *A. urmiana* is well illustrated and precise (Fig. 7).

The synonymic list (synonyms and new combinations) for the Western Asian Lineage remains as follows:

***Artemia urmiana*** Günther, 1899

*Branchipus milhausenii* Fischer de Waldheim, 1834: 459 (*nomen oblitum*). **New synonymy**. Terra typica: Not stated in the original description, but a couple of pages earlier, Fischer de Waldheim (1834: 457) indicated that Milhausen found the species “dans le lac salé Sak en Crimée”. Only female specimens were mentioned or described in the original work.

*Artemia mulhausenii* (Fischer de Waldheim, 1834): Milne-Edwards, 1840: 370. Many authors considered that Fischer (1851: 155) described a new species of *Artemia* under the name *Artemia muellhausenii* Fischer, 1851. However, Fischer (1851) clearly indicated that his description was intended only to improve former descriptions of the same taxon by Fischer de Waldheim (1834 sub *Branchipus milhausenii*) and Rathke (1836 sub *A. salina)*, both made using materials from Crimea. Fischer (1851) used for Fischer de Waldheim (1834) taxon, the spelling modified by Milne-Edwards (1840), adding an extra-l.

*Artemia koeppeniana* Fischer, 1851: 157 (*nomen oblitum*). **New synonymy**. Terra typica: “… im südlichen Russland gesammelt,”. Only female specimens mentioned (Fischer, 1851).

*Branchipus koeppenianus* (Fischer, 1851): Grube, 1853: 140.

*Artemia proxima* King, 1855: 70 (*nomen oblitum*). **New synonymy**. Terra typica: “Salt Pans, Newington; Parramatta”. Types not indicated (King, 1855, 1866).

*Artemia salina var. biloba* Entz, 1886: 105 (*nomen oblitum*). **New synonymy**. Terra typica: “Ezen fajta a tömör sótartalmú tavakat, Vizaknan a 20%—os Tökölyit, Tordán a 10%—os Aknafürdőt lakja.” [Romania: Transylvania: Lake Tökölyit in Vizaknán (Ocna Sibiului), Lake Aknafürdőt in Tordán (Turda)]. Type not designated. Only female populations (Entz, 1886). Daday de Deés (1910) established its synonymy with *A. salina* var. *milhausenii*.

*Artemia salina var. furcata* Entz, 1886: 106 (*nomen oblitum*). **New synonymy**. Terra typica: “Ezen fajta a hígabb sotartalmú tavakat, Vizaknan a 7.65%—os Vörös-, és Asszonytavat, Tordán a Banyafürdő 4%—os tavait lakja.” [Romania: Transylvania: Lake Vörös and Asszonytavat in Vizaknán (Ocna Sibiului), Lake Banyafürdő in Tordán (Turda)]. Type not designated. Only female populations (Entz, 1886). Daday de Deés (1910) established its synonymy with *A. salina* var. *arietina*.

*Artemia asiatica* Walter, 1887: 926 (*nomen oblitum*). **New synonymy**. Terra typica: “In einer Salzquelle zwischen Bend-i-nadyr und dem Brunnen Agamet in der Bergwüste östlich vom Murgab, nahe der Afghanengrenze”. The type series includes only females (Walter, 1887). Walter (1888a, 1888b) indicated that only female specimens were collected in a salt-spring in the hillside of the Afghan border, east of Saryken-Aul at Murgab [Bãlã Morgãb, 35°38′N–63°18′, Afghanistan, see map in Radde & Walter (1889), between Bend-i-nadyr [ca. 35°51′N–63°07′E] and the desert-well Agamet, on 14–26 April 1887, a salty lake with thickened edges by precipitated salt.

*Artemia urmiana* Günther, 1899: 395 (*nomen protectum*). Terra typica: “Lake Urmi, in water of specific gravity 1.1138.”. Bisexual (Günther, 1899; Fig. 7). Barigozzi et al. (1988) failed to find bisexual populations at Lake Urmia, and found only parthenogenetic populations.

? *Artemia australis* Sayce, 1903: 229 (*nomen dubium*). Terra typica: “Brackish-water, Sandhills, Gleneg, coastal district of South Australia…”. Sayce (1903) stated that over 100 specimens all were females, and that the large number of young forms observed probably were of parthenogenetic origin.

? *Artemia westraliensis* Sayce, 1903: 230 (*nomen dubium*). Terra typica: “Lake Aurean, Murchison, West Australia…”. The type series consists of two female specimens (Sayce, 1903).

*Artemisia proxima* (King, 1855): Dakin, 1914: 294.

*Artemisia australis* (Sayce, 1903): Dakin, 1914: 294.

*Artemisia westraliensis* (Sayce, 1903): Dakin, 1914: 296.

*Artemia parthenogenetica* Bowen & Sterling, 1978: 595. **New synonymy.** Terra typica: not stated explicitly, but the authors included five parthenogenetic populations in its category: “… Madras and Kutch, India; Port Hedland, Australia; Sète, France; and Yamaguchi-ken, Japan”. Type series or type material not designated, and ploidy not stated (Bowen & Sterling, 1978). It is very possible that materials used in this work included both 2n and 4n parthenogenetic populations, but most of the populations included by Bowen & Sterling (1978) correspond today to the *A. urmiana* clade (Triantaphyllidis, Abatzopoulos & Sorgeloos, 1998; Muñoz & Pacios, 2010). A lectotype designation (if type specimens exist) or neotype (if they are lost) is necessary to assure the correct synonymization of this name.

? *Artemia barkolica* Qian & Wang in Qian et al. (1992) (*nomen dubium*). Terra typica according to Asem et al. (2020): Barkol Lake, Xinjiang, China. Male and female specimens known. Specimens from Barkol Lake were studied at the molecular level by Asem, Eimanifar & Sun (2016); these sequences are nested within the Western Asian Lineage. Asem et al. (2020) already considered this taxon to be composed of several phylogenetic clades (all present in this location), but they did not provide a formal statement on its synonymy.

*Artemia urumuqinica* Qian & Wang in Qian et al. (1992) (*nomen dubium*). Terra typica according to Asem et al. (2020): Urumqi Caiwuo Pu Yan Hu, Xinjiang, China. Only female specimens known. Asem et al. (2020) considered it a possible synonym of previously described taxa, but they did not provide any formal statement on its synonymy.

*Artemia ebinurica* Qian & Wang in Qian et al. (1992). **New synonymy**. Terra typica according to Asem et al. (2020): Ebinur, Xinjiang, China. Male and female specimens known. *Cox1* sequences of specimens from Aibi Lake were studied at the molecular level by Maccari et al. (2013) and Asem, Eimanifar & Sun (2016); these sequences are included within the Western Asian Lineage. Asem et al. (2020) mentioned that they could be considered as synonyms of previously described taxa, but they did not provide any formal statement on its synonymy.

*Artemia tibetiana* Abatzopoulos, Zhang & Sorgeloos, 1998: 43. **New synonymy**. Terra typica: “… in Lagkor Co Lake on the high plateaus of Tibet (P.R. China).” “Lagkor Co is a carbonate lake, situated 4,490 m above sea level in the arid-temperate plateau zone of Tibet, at 84° 13′ E and 32° 03′ N… ”. Types not designated. Bisexual (Abatzopoulos, Zhang & Sorgeloos, 1998). The taxon was characterized by presenting large cyst diameter (323 µm + 17.2; 330 µm +14.6), the largest length of first instar nauplii (667 µm + 32.7), and the largest adult size recorded among *Artemia* species (Abatzopoulos, Zhang & Sorgeloos, 1998). However genetic divergence based on allozyme analyses, and reproductive incompatibility (postzygotic isolation) between this Tibetan populations and other Asian populations studied was relatively low (allozymes), or not significantly different (fertility) than those obtained for intraspecific crossings (Abatzopoulos, Zhang & Sorgeloos, 1998).

*Artemia murae* Naganawa in Naganawa & Mura (2017): 1684. **New synonymy**. Terra typica: “Tonkhil nuur (Tonkhil Lake), Tonkhil sum., Gobi-Altai aimag, Mongolia (46°10′10″N 93°55′00″E),…”. Bisexual. This population deserves further molecular analyses (see above).

*Artemia frameshifta* Naganawa & Mura, 2017: 1688. **New synonymy**. Terra typica: “Bajan-Onjul, Tov aimag, Mongolia…”. Only female specimens known.

### Eastern Asian Lineage—*Artemia sinica*

The Eastern Asian Lineage is composed of two to three relatively well supported *cox1* mtDNA sister clades. The available information on nuclear markers, suggests that either gene flow is still ongoing across them, or that actual isolation across mtDNA clades is so recent that there is no evidence of nuclear isolation (Baxevanis, Kappas & Abatzopoulos, 2006; Eimanifar et al., 2014; Asem, Eimanifar & Sun, 2016). Again, divergence among mtDNA clades within the Eastern Asian Lineage is low compared to what is found among old mtDNA clades in other species of *Artemia*, for which the existence of gene flow across mtDNA clades has been demonstrated to occur (Eimanifar et al., 2014). Consequently, all populations structured in *cox1* clades within the Eastern Asian Lineage should be treated as a single evolutionary and taxonomic unit.

The Eastern Asian Lineage includes bisexual and 4n–5n polyploid parthenogenetic specimens (Baxevanis, Kappas & Abatzopoulos, 2006; Asem, Eimanifar & Sun, 2016). Bisexual populations are morphologically diagnosable (Cai, 1989a, 1989b), while bisexual and parthenogenetic populations are genetically characterized with respect to all other lineages (Baxevanis, Kappas & Abatzopoulos, 2006), but see Eimanifar et al. (2014) and Asem, Eimanifar & Sun (2016) to get an idea of the large diversity shown by rapidly evolving nuclear data. Mitochondrial DNA variability within either parthenogenetic or bisexual populations is very limited (Naihong et al., 2000). All 4n and 5n parthenogenetic specimens cluster in a single clade (based on *cox1*, *12S* and *16S mtDNA*), related but not nested within the bisexual clade from China (Maniatsi et al., 2011; Asem, Eimanifar & Sun, 2016). *Cox1* divergence between the 4n and 5n parthenogenetic clade and the bisexual populations is relatively large (Maniatsi et al., 2011; Asem, Eimanifar & Sun, 2016), suggesting that parthenogenesis and polyploidy arose after separation of the two clades. Nuclear data, either *ITS1* or slow evolving nuclear genes, such as *Na*^*+*^*/K*^*+*^ *ATPase*, show that some specimens of 4n populations share alleles with 2n and bisexual populations of the Western Asian Lineage, while all other 4n and 5n display a wide array of alleles some of them related to bisexual populations of the Eastern Asian Lineage (Baxevanis, Kappas & Abatzopoulos, 2006; Asem, Eimanifar & Sun, 2016). However, nuclear data of parthenogenetic polyploid populations are of difficult interpretation since polyploidy generates multiple nuclear copies.

Parthenogenesis and polyploidy are strong speciation factors when sufficient time is provided, sometimes leading to complete isolation and the formation of independent taxa (Chaplin & Hebert, 1997; Mark Welch & Meselson, 2000; Cunha, Doadrio & Coelho, 2008; but see Hurst & Peck, 1996; Schön, Martens & Rossi, 1996). In parthenogenetic *Artemia*, each polyploidy event could be treated as a speciation event, resulting thus in multiple agamospecies (Mayr, 2001) as indicated by Maniatsi et al. (2011). Despite the time elapsed from their split from the bisexual Eastern Asian Lineage, all 4n specimens studied so far share a common *cox1* haplotype, with only one mutation step minor variants (Baxevanis, Kappas & Abatzopoulos, 2006; Maniatsi et al., 2011), consequence of very recent mutation events, or more likely derived from sequence reading problems or PCR noise. This implies that 4n parthenogenetic populations were originated very recently from a bisexual ancestral population sister to the bisexual populations of the Eastern Asian Lineage. Therefore, even if current tetraploid parthenogenetic populations could be isolated (Maniatsi et al., 2011), their hypothetical recent bisexual ancestor is likely not. We again agree with Baxevanis, Kappas & Abatzopoulos (2006), Maniatsi et al. (2011), and Eimanifar, Van Stappen & Wink (2015) in considering that these 4n and 5n parthenogenetic populations are part of an Eastern Asian Lineage, and they should not be treated as an independent taxon from the bisexual populations that originated them.

Only one species name, *Artemia sinica* Cai, 1989, has been applied with certainty to the Eastern Asian Lineage in addition to *A. parthenogenetica* (in part). However, it could be possible that some of the *nomina dubia* included tentatively under *A. urmiana*, corresponded in fact to the Eastern Asian Lineage (see comments in Asem et al., 2020). Then, some of those names might have priority over *A. sinica*. The description of *A. sinica* by Cai (1989a) was published in a short format and latter corrected and completed, with better quality images (Cai, 1989b).

So far, the synonymic list for the Eastern Asian Lineage remains as follows:

***Artemia sinica*** Cai, 1989

*Artemia sinica* Cai, 1989a: 40. Terra typica: “…from the 150 km2 Xie-chi sulphate salt lake, located east of the city of Yun Chang in the Shan-xi Province in Central China.”. Bisexual (Cai, 1989a, 1989b). The figure presented in Cai (1989a) was published in better quality in Cai (1989b). Cai (1989b) corrected some data of the type locality: Yun Cheng salt lake, Shanxi Province, China; and described the morphologically differential characters. Cai (1989b) indicated that the species presents 42 chromosomes and that it is reproductively isolated from the rest of species in the genus and is morphologically distinguishable from all other bisexual species.

### Remarks on the morphology of *Artemia*

Many authors have studied different aspects of the morphology of *Artemia*, including qualitative and quantitative traits and its state at different moments of development (Schmankewitsch, 1873, 1877b; Artom, 1907a; Abonyi, 1915; Gilchrist, 1960; Tyson & Sullivan, 1979, 1980; Amat, 1980a, 1980b; Wolfe, 1980; Schrehardt, 1987; Mura, Del Caldo & Fanfani, 1989; Mura, Fanfani & Del Caldo, 1989; Mura, 1990; Hontoria & Amat, 1992a, 1992b; Mura & Del Caldo, 1992; Torrentera & Dodson, 1995; Brendonck & Belk, 1997; Triantaphyllidis et al., 1997a, 1997b; Gajardo et al., 1998; Cohen et al., 1999; Cohen, Rodríguez Gil & Vélez, 1999; Zúñiga et al., 1999; Mayer, 2002; Torrentera & Belk, 2002; Mura & Brecciaroli, 2004; Abatzopoulos et al., 2009; Baxevanis et al., 2005; Asem, Rastegar-Pouyani & Agh, 2007; Asem & Rastegar-Pouyani, 2008; Asem et al., 2010; De Los Ríos & Asem, 2008; Asem & Rastegar-Pouyani, 2010; Vetriselvan & Munuswamy, 2011; Naceur, Jenhani & Romdhane, 2012, 2013; Asem & Sun, 2016). Most of them concluded that inter-populational variability is so high as to impede using the characters studied for species discrimination unless specimens are reared at controlled laboratory conditions (Mura & Brecciaroli, 2004; Abatzopoulos et al., 2009; Asem et al., 2010). Temperature and ionic composition and concentration were mainly responsible for the differences found among populations from close locations or among seasonal cohorts in a single location (Schmankewitsch, 1877b; Abonyi, 1915; Amat, 1980b; Naceur, Jenhani & Romdhane, 2012).

Some quantitative characters, including abdominal length, size and shape of the ovisac, length of the furca, number of setae on furcal branches, and size and shape of head appendages, as eye diameter and length of the antenna, have been shown to enable taxon discrimination when specimens are reared under similar developmental conditions (Hontoria & Amat, 1992a, 1992b). In this situation, Baxevanis et al. (2005) reported that both sexes of bisexual *A. urmiana* can be differentiated from *A. sinica* and *A. monica* (= *A. franciscana)* based on the display of a very thin and long abdomen, the shape of the ovisac, and the remarkable short furcal branches, which either have few setae or are completely naked. *Artemia sinica* differs from representatives of the *A. monica* clade in the relative length/width ratio of the abdomen (Cai, 1989b). In *A. persimilis* each of the furcal rami of adults bears three to five feathered setae (Cohen et al., 1999), while adults of *A. monica* bear generally 12 to 15 each (Schrehardt, 1987 sub *A. franciscana*).

Qualitative characters such as shape, size and ornamentation of the frontal knobs of male antennae, and presence and ornamentation of spine-like projections at the base of penises, are reliable features for the identification of *A. persimilis* and *A. salina*, but are less useful for the recognition of other taxa (Triantaphyllidis et al., 1997a; Mura & Brecciaroli, 2004; Abatzopoulos et al., 2009). The frontal knob of the male antenna is sub-spherical, large and poorly ornamented in *A. persimilis*, it is sub-cylindrical in *A. salina*, whereas it is sub-spherical but smaller and covered with dense papillae in specimens of *A. monica*, *A. urmiana* and *A. sinica* (Mura & Brecciaroli, 2004). Ornamentation of the basal spines of penis (absent in *A. salina*) can be used to separate *A. persimilis* from all other species: *A. persimilis* presents a few tooth-like protuberances scattered on the surface, while specimens of *A. monica*, *A. urmiana* and *A. sinica* present a dense cover of scale-like projections covering the tip of each penis (Mura & Brecciaroli, 2004).

Adult males of *A. salina* are characterized by the display of sub-cylindrical frontal knobs, which are sub-spherical in all other species, and by the absence of a basal spine on the penises, (vs. present in all other species). The sub-spherical frontal knob of the male antennae of *A. sinica* is generally smaller than those of specimens of *A. monica* (= *A. franciscana*) and *A. urmiana* (Cai, 1989a, 1989b).

### Identification key to males of *Artemia*

Note that separation between specimens of *A. monica* (= *A. franciscana*), *A. sinica* and *A. urmiana* cannot be established with certainty unless specimens are reared under controlled conditions. Morphological characters used in the key were mainly obtained from Cai (1989b), Triantaphyllidis et al. (1997a), Cohen et al. (1999), Mura & Brecciaroli (2004), and Baxevanis et al. (2005).

1. Penises without spine outgrowth on the basal part; antennal frontal knobs sub-cylindricalArtemia salina
  Penises with spine outgrowth on the basal part; antennal frontal knobs sub-spherical2
2. Basal spine of the penises without terminal scale-like projections and with a few tooth-like protuberances scattered on surface; frontal knobs large and poorly ornamentedArtemia persimilis
  Scale-like, acute, projections covering completely the apical end of the basal spine of the penises; small, densely ornamented frontal knobs with spines and setae3
3. Abdomen proportionally long, furcal branches remarkably short, rami with few to none plumose setaeArtemia urmiana
  Abdomen proportionally short, furcal branches each with less than 15 plumose setae4
4. Frontal knobs large, with large basis; abdominal segments proportionally broad*Artemia monica* (= *A. franciscana*)
  Frontal knobs small, with small basis; abdominal segments proportionally slenderArtemia sinica

## Conclusions

The proper names for the evolutionary units in which brine shrimps are structured remain as follows: *Artemia persimilis* Piccinelli & Prosdocimi, 1968 for the Southern Cone Lineage; *Artemia salina* (Linnaeus, 1758) for the Mediterranean-South African Lineage; *Artemia monica* Verrill, 1869 (= *A. franciscana* Kellogg, 1906) for the New World Lineage *Artemia urmiana* Günther, 1899 for the Western Asian Lineage; and *Artemia sinica* Cai, 1989 for the Eastern Asian Lineage.

Future research to identify species-level lineages in *Artemia* is still required in different geographic areas. The Mediterranean and South African populations of *A. salina* are so distant geographic and genetically that they could represent two independent taxonomic units (Muñoz et al., 2008; but see Baxevanis et al., 2014). The mtDNA phylogeographic structure within *A. monica* (= *A. franciscana*), depicts a series of relatively isolated units (Puerto Rico, México among others, see Fig. 5) so separated from the remaining ones, that deserve a detailed nuclear study to set the level of gene flow among them. The extent of gene flow occurring among the different Tibetan populations and also with respect to other Asian populations needs to be revised. Since the population of *A. tibetiana* from the type locality shows a relatively high-level of gene exchange with Asian populations of *A. urmiana* (Baxevanis, Kappas & Abatzopoulos, 2006; Eimanifar et al., 2014; Asem, Eimanifar & Sun, 2016), its synonymy seems to be justified. But, other Tibetan populations might not be subjected to equal amounts of gene flow, and could represent undescribed taxa (Kappas, Baxevanis & Abatzopoulos, 2011; Eimanifar et al., 2014). Finally, bisexual Hawaiian (Sars, 1904) and coastal Peruvian (Grube, 1874) populations, bisexual and parthenogenetic Chinese populations (Qian et al., 1992), and parthenogenetic populations from Australia (Sayce, 1903), all require of molecular data to guarantee a precise identification to confirm their synonymy with other published names.

## Appendix I

### *Nomina nuda* and other unavailable names in *Artemia*

A *nomen nudum* is “*a name that, if published before 1931, fails to conform to Article 12; or, if published after 1930, fails to conform to Article 13. A nomen nudum is not an available name, and therefore the same name may be made available later for the same or a different concept; in such a case it would take authorship and date [Arts. 50, 21] from that act of establishment, not from any earlier publication as a nomen nudum*.” (International Commission on Zoological Nomenclature, 1999). Article 12 explicitly indicates that: *“To be available, every new name published before 1931 must satisfy the provisions of Article 11 and must be accompanied by a description or a definition of the taxon that it denotes, or by an indication*”. Article 13 explicitly indicates: “*T. be available, every new name published after 1930 must satisfy the provisions of Article 11 and must 13.1.1. be accompanied by a description or definition that states in words characters that are purported to differentiate the taxon, or 13.1.2. be accompanied by a bibliographic reference to such a published statement, even if the statement is contained in a work published before 1758, or in one that is not consistently binominal, or in one that has been suppressed by the Commission (unless the Commission has ruled that the work is to be treated as not having been published [Art. 8.7]), or 13.1.3. be proposed expressly as a new replacement name (nomen novum) for an available name, whether required by any provision of the Code or not*.”

According to the International Commission on Zoological Nomenclature (1999) criteria for a name to be considered a *nomen nudum* (Articles 12 and 13 of the Code), none of the following *Artemia* names are *nomina nuda*: *Eulimene albida* Latreille, 1816; *Branchipus milhausenii* Fischer de Waldheim, 1834; *Artemia salina* f. *arietina* Fischer, 1851; *Artemisia proxima* King, 1855; *Branchipus oudneyi* Liévin, 1856; *Artemia jelskii* Grube, 1874; *Artemia salina* var. *biloba* Entz, 1886; *Artemia salina* var. *furcata* Entz, 1886; *Callaonella dybowski* Grochowski, 1896; *Artemia westraliensis* Sayce, 1903; and *Artemia salina* var. *pacifica* Sars, 1904. They are all available names.

However, “*Artemia elegans* Seale, 1933”, “*Artemia americana* Barigozzi, 1974”, “*Artemia odessensis* Barigozzi, 1980”, “*Artemia sinica aibihuensis* Yin, Zhang & You, 2013”, “*Artemia sinica gahaiensis* Yin, Zhang & You, 2013”, “*Artemia sinica jingyuhuensis* Yin, Zhang & You, 2013”, and “*Artemia sinica xiaochaidanensis* Yin, Zhang & You, 2013”, meet the requirements to be considered *nomina nuda* and therefore are unavailable (Asem et al., 2020; see also Article 11.5).

All other names from Simon (1886), Samter & Heymons (1902), Artom (1906a, 1906b, 1906c, 1912, 1921b), and Perrier (1929), included by Rogers (2013) as *nomina nuda*, and those included by Vikas et al. (2012) (Asem et al., 2020) rather correspond to denominations that the authors never intended to become taxonomic nomenclatural acts, or to names that actually were never used by them. Artom (1905, 1906a, 1906b, 1906c, 1907a, 1907b, 1908, 1911, 1912, 1913, 1921a, 1921b, 1922, 1924, 1926, 1931) performed a series of meticulous experiments demonstrating the existence of an ovoviviparous reproductive mode, and the presence of parthenogenetic and also tetraploid populations of *Artemia*, which he considered to be differentiated species. However, Artom (op. cit.) never intended to provide new names for these species or describing them (Bond, 1934); contrary to the opinion of Barigozzi (1974, 1980). Artom (op. cit.) used names as biological terms, in Italian, referring to the biological traits of the populations he was studying: “Artemie sessuate”, “Artemie partenogenetiche”, “*Artemia* di Cagliari”, “*Artemia* partenogenetica di Capodistria”, “varietà sessuata”, “forma sessuata”, “*Artemia* sessuata”, “varietà partenogenetica”, “Artemie partenogenetiche di Marsiglia e di Capodistria”, “*Artemia* a partenogenesi indefinita”, “*Artemia* sessuata di Cagliari”, “*Artemia sessuata* di Cagliari”, “*Artemia salina sessuata* di Cagliari”, “*Artemia partenogenetica* di Capodistria”, “*Artemia salina partenogenetica* di Capo d’Istria”, “*Artemia salina* di Capodistria”, “Artemia *micropirenica*”, “Artemia *macropirenica*”, “Artemie *micropireniche*”, “Artemie *macropireniche*”, “*Artemia salina* partenogenetica di Odessa”, “*Artemia partenogenetica* di Odessa”, “Artemie (*univalens* di Cagliari e *bivalens* di Capo d’Istria)”, “*Artemia bivalens*”, “*Artemia univalens*”, “specie univalens sessuata (Cagliari)”, specie “bivalens partenogenetica (Capodistria e Odessa)”, “*Artemia salina univalens*”, “*Artemia salina bivalens*”, “*Artemia salina bivalens* di Capo d’Istria”, “*Artemia salina* di Cagliari (*univalens*)”, “*Artemia salina* di Capo d’Istria (*bivalens*)”, “*Artemia salina bivalens* di Odessa” (Artom, op. cit.; italics as in the original). These adjectives, used in different forms in the same page, are not taxonomic actions and are not available for nomenclatural purposes (Art. 1.3.5). Therefore, they cannot be included in the synonymy of any species of *Artemia* (Belk & Brtek, 1995), nor treated as *nomina nuda* (Rogers, 2013). A similar situation occurs with Samter & Heymons (1902) diverse expresions; however, Samter & Heymons (1902) never used a name such as “*Artemia cagliaritana*”, wrongly attributed to them in various synonymic lists (*nomen dubium* according to Belk & Brtek (1995), and *nomen nudum* according to Rogers (2013 sub “*Artemia cagliartiana*”), or to Barigozzi (1980: 150 sub “*Artemia calaritana*”)). However, Artom (1905: 286) did use such a name to indicate that there is no justification to legitimate the creation of a new species: “… non sono tali da legittimare la creazione di una nuova specie di *Artemia Cagliaritana*”, obviously referring to the geographic location of his samples, as stated a few lines below: “…di una especie *Cagliaritana*”. These words cannot be considered a nomenclatural act. In a similar manner, Simon (1886) subdivision of morphotypes in *A. salina*: “Forma principalis”, “Forma intermedia”, “Forma Milhauseni”, and “Forma Köppeniana”, were not intended to be used as taxonomic entities. However, Daday de Deés (1910) made the name *A. salina* var. *principalis* available. Barigozzi (1974, 1980) with an evident disregard for taxonomic nomenclature, complicated matters by proposing infrasubspecific and new (unavailable) names and by considering Artom’s (op. cit.) descriptions, nomenclatural acts.

In summary, the following names mentioned by previous authors (Belk & Brtek, 1995; Rogers, 2013) are unavailable for taxonomic nomenclature purposes and consequently should be stated as such, or simply should not to be placed in the synonymic list of any species of *Artemia*: “*Artemia salina f. intermedia* Simon, 1886”; “*Artemia salina f. principalis* Simon, 1886”; “*Artemia cagliaritana* Samter & Heymons, 1902”; “*Artemia salina partenogenetica* Artom, 1906”; “*Artemia salina sessuata* Artom, 1912”; “*Artemia salina univalens* Artom, 1912”; “*Artemia salina bivalens* Artom, 1912”; “*Artemia bivalens partenogenetica* Artom, 1912”; “*Artemia micropirenica* Artom, 1921”; “*Artemia salina f. typica* Perrier, 1929”; “*Artemia elegans* Seale, 1933; *Artemia americana* Barigozzi, 1974”; “*Artemia odessensis* Barigozzi, 1980”; “*Artemia kazakhastan* Vikas et al., 2012”; and “*Artemia china* Vikas et al., 2012”. See Asem et al. (2020) for details with respect for these two last names.

Some additional names, “*Artemia salina var. arietina f. brachycerca* Daday de Deés, 1910”; “*Artemia salina var. arietina f. dolichocerca* Daday de Deés, 1910”; “*Artemia salina var. arietina f. eurycerca* Daday de Deés, 1910”; “*Artemia salina var. arietina f. oligotricha* Daday de Deés, 1910”; “*Artemia salina var. arietina f. polytricha* Daday de Deés, 1910”; and “*Artemia salina var*. *principalis* Daday de Deés, 1910”, were used to describe intrapopulational variation and infrasubspecific taxa and therefore, are also unavailable according to the International Commission on Zoological Nomenclature (1999).

Once all these aforementioned names are removed from consideration, there are still 31 available names that could be applied to taxa within *Artemia* (see main text and “Appendix II”).

## Appendix II

### *Nomina dubia* in *Artemia*

A *nomen dubium* is “*a name of unknown or doubtful application*”, but nonetheless, an available name (International Commission on Zoological Nomenclature, 1999). There are many names of doubtful application in *Artemia* (Belk & Brtek, 1995). The incorporation of reproductive biology and molecular data to the current species concepts, makes very difficult the assignation of the older and some recent names to the taxon they belong to.

Names falling within the category of *nomina dubia* are: *Eulimene albida* Latreille, 1816, “…dans la Méditerranée…”, type locality not precise and reproductive mode not indicated (Latreille, 1816). *Artemia eulimene* Leach, 1819, “Habite la Méditerranée, près Nice”, type locality precise, but reproductive mode not indicated (Leach, 1819). *Artemia proxima* King, 1855, “Salt Pans, Newington; Parramatta”, type locality impossible to locate (according to Sayce (1903)), but currently Newington and Parramatta are suburbs of Sydney (New South Wales); Newington produced large quantities of salt along the 19th century; in case the population still exists, it requires DNA data. *Artemia gracilis* Verrill, 1869, “Near New Haven, in tubs of water from salt marsh”, type locality precise, but not found again; if the population still exists requires DNA data. *Artemia jelskii* Grube, 1874, “…Callao…”, it is possible that the specimens studied were originated from any saltern along the coast of Perú, but shipped to Europe from Callao, the main commercial port at the time in the area; if the population still exists it would be necessary to study its genetic identity. *Artemia australis* Sayce, 1903, “Brackish-water, Sandhills, Glenelg, coastal district of South Australia…”, probably parthenogenetic based on the material studied originally (Sayce, 1903), requires DNA data. *Artemia westraliensis* Sayce, 1903, “Lake Aurean, Murchison, West Australia…”, probably parthenogenetic based on the type series (Sayce, 1903); if the population still exists requires DNA data. *Artemia barkolica* Qian & Wang, 1992, sequences are nested within the Western Asian Lineage, but there are contradictory data on the reproductive mode of this population (Asem et al., 2020). *Artemia urumuqinica* Qian & Wang, 1992, is likely a synonym of *A. urmiana*, but more clarifying data are necessary. For these names, we can only make a tentative attempt of species allocation.

## Supplemental Information

10.7717/peerj.10865/supp-1 Supplemental Information 1 Circular map of the *Artemia* mitogenome. The abbreviations for the genes are as follows: *cox1*, cox2, and cox3 refer to the cytochrome C oxidase subunits; CytB refers to cytochrome B; and nad1–6 refers to NADH dehydrogenase subunits; atp6 and atp8 refer to subunits 6 and 8 of F0 ATPase; rrnL and rnnS refer to the 16s and 12S rRNA genes. The major non-coding region, D-loop and associated promoters (DLP), is shown in grey. Arrows indicate the direction of transcription. Protein coding genes are depicted in green, tRNAs in pink and ribosomal RNA in red.

10.7717/peerj.10865/supp-2 Supplemental Information 2 Chronogram showing lineage divergence times in *Artemia* obtained using BEAST following the second scenario hypothesis (Scheme 2). Chronogram showing lineage divergence times in *Artemia* obtained using BEAST following the second scenario hypothesis (Scheme 2). Time indicated in million years (Ma). Dark blue horizontal bars represent 95% HPD (High Posterior Density). A posterior probability value of 1 was obtained for all nodes.

10.7717/peerj.10865/supp-3 Supplemental Information 3 Chronogram showing lineage divergence times in *Artemia* obtained using BEAST following the third scenario hypothesis (Scheme 3). Chronogram showing lineage divergence times in *Artemia* obtained using BEAST following the third scenario hypothesis (Scheme 3). Time indicated in million years (Ma). Dark blue horizontal bars represent 95% HPD (High Posterior Density). A posterior probability value of 1 was obtained for all nodes.

10.7717/peerj.10865/supp-4 Supplemental Information 4 Chronogram showing lineage divergence times in *Artemia* obtained using BEAST following the fourth scenario hypothesis (Scheme 4). Chronogram showing lineage divergence times in *Artemia* obtained using BEAST following the fourth scenario hypothesis (Scheme 4). Time indicated in million years (Ma). Dark blue horizontal bars represent 95% HPD (High Posterior Density). A posterior probability value of 1 was obtained for all nodes.

10.7717/peerj.10865/supp-5 Supplemental Information 5 Annotations of the mitogenome content of *A. salina*.. Mitochondrial DNA contains 13 protein genes, two rRNA genes and 22 tRNA genes. Length, size, positions, start/stops codons (protein genes) and anticodons (tRNA) are specified per gene. DLP stands for D-loop and associated promoters. *TAA stop codon is completed by the addition of 3’ A residues to the mRNA.

10.7717/peerj.10865/supp-6 Supplemental Information 6 Annotations of the mitogenome content of *Artemia franciscana*. Annotations of the mitogenome content of *Artemia franciscana*. Mitochondrial DNA contains 13 protein-coding genes, two rRNA genes and 22 tRNA genes. Length, size, positions, start/stops codons (protein genes) and anticodons (tRNA) are specified per gene. DLP stands for D-loop and associated promoters. *TAA stop codon is completed by the addition of 3’ A residues to the mRNA.

10.7717/peerj.10865/supp-7 Supplemental Information 7 Number of mitogenome partitions (subsets) in which protein and ribosomal genes were structured and their best estimated evolutive models in order to perform the phylogenetic reconstruction under the Bayesian Information Criterion.

## Additional Information and Declarations

### Field Study Permissions

The following information was supplied relating to field study approvals (i.e., approving body and any reference numbers): Field permits were granted by SEMARNAT (México) (oficio n° SGPA-DGVS-04386-15).

### Data Availability

The following information was supplied regarding data availability: Data are available at NCBI: MT495440, MT495441. *A. salina* and *A. monica* sequences are now available at GenBank: SAMN16079497 and SAMN16079498, respectively.
