# Supplementary figures and images for: Settling taxonomic and nomenclatural problems in brine shrimps, Artemia (Crustacea: Branchiopoda: Anostraca), by integrating mitogenomics, marker discordances and nomenclature rules
Source: PeerJ. 2021 Mar 10;9:e10865. doi: 10.7717/peerj.10865 (PMC7955675; doi:10.7717/peerj.10865)

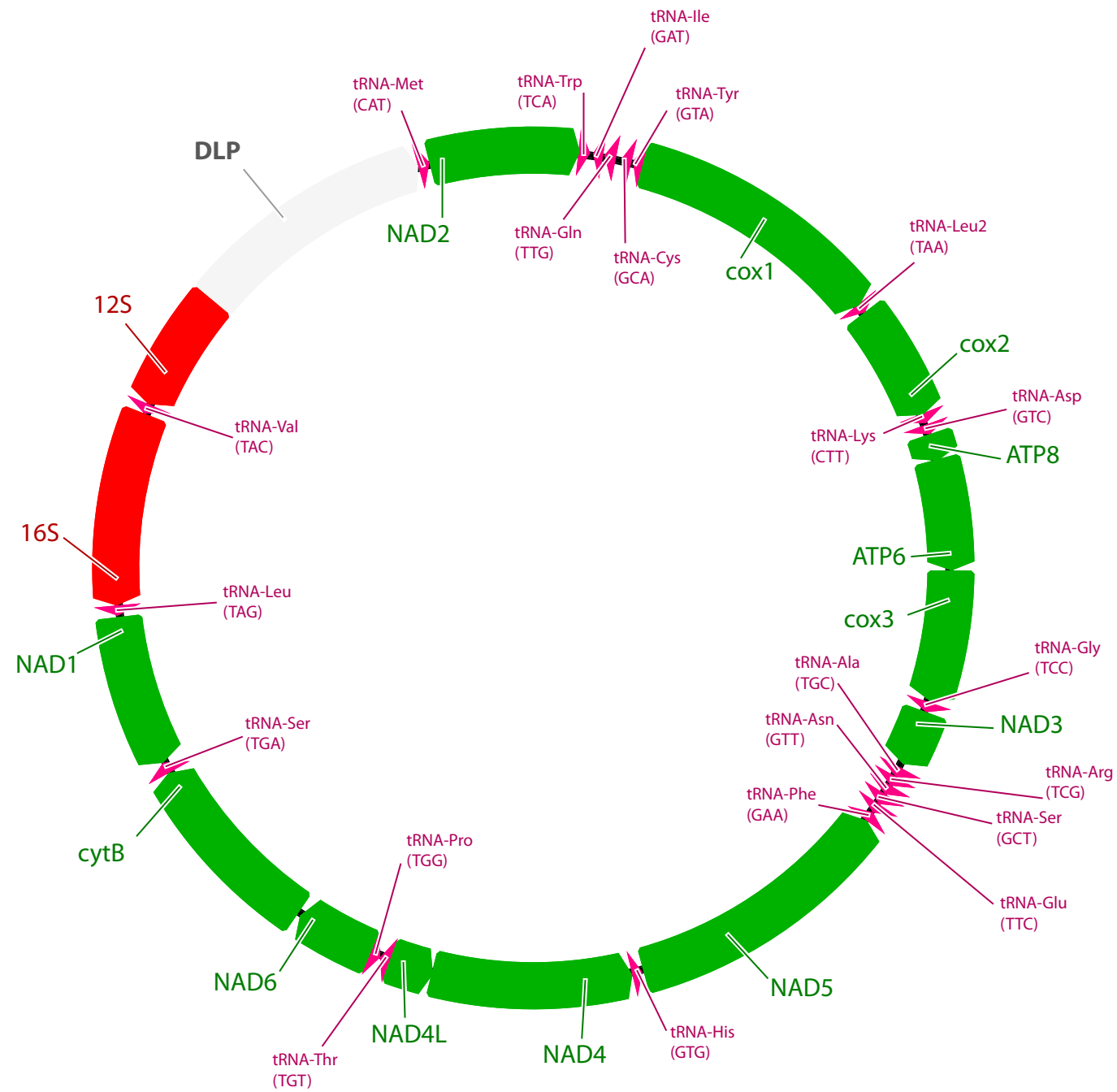

Supplement: Supplemental Information 1 — The abbreviations for the genes are as follows: cox1, cox2, and cox3 refer to the cytochrome C oxidase subunits; CytB refers to cytochrome B; and nad1–6 refers to NADH dehydrogenase subunits; atp6 and atp8 refer to subunits 6 and 8 of F0 ATPase; rrnL and rnnS refer to the 16s and 12S rRNA genes. The major non-coding region, D-loop and associated promoters (DLP), is shown in grey. Arrows indicate the direction of transcription. Protein coding genes are depicted in green, tRNAs in pink and ribosomal RNA in red. [file peerj-09-10865-s001.pdf]

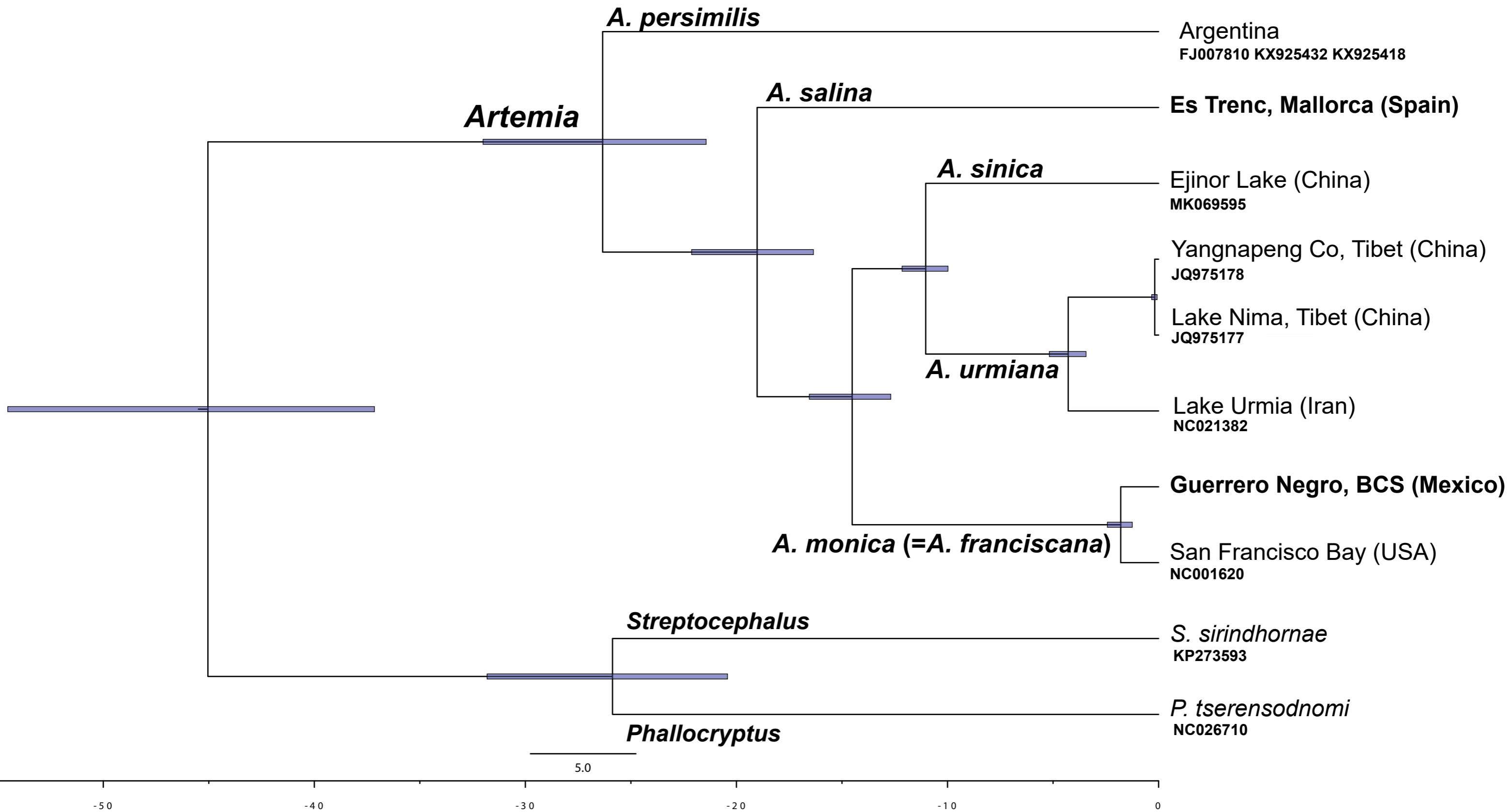

Supplement: Supplemental Information 2 — Chronogram showing lineage divergence times in Artemia obtained using BEAST following the second scenario hypothesis (Scheme 2). Time indicated in million years (Ma). Dark blue horizontal bars represent 95% HPD (High Posterior Density). A posterior probability value of 1 was obtained for all nodes. [file peerj-09-10865-s002.pdf]

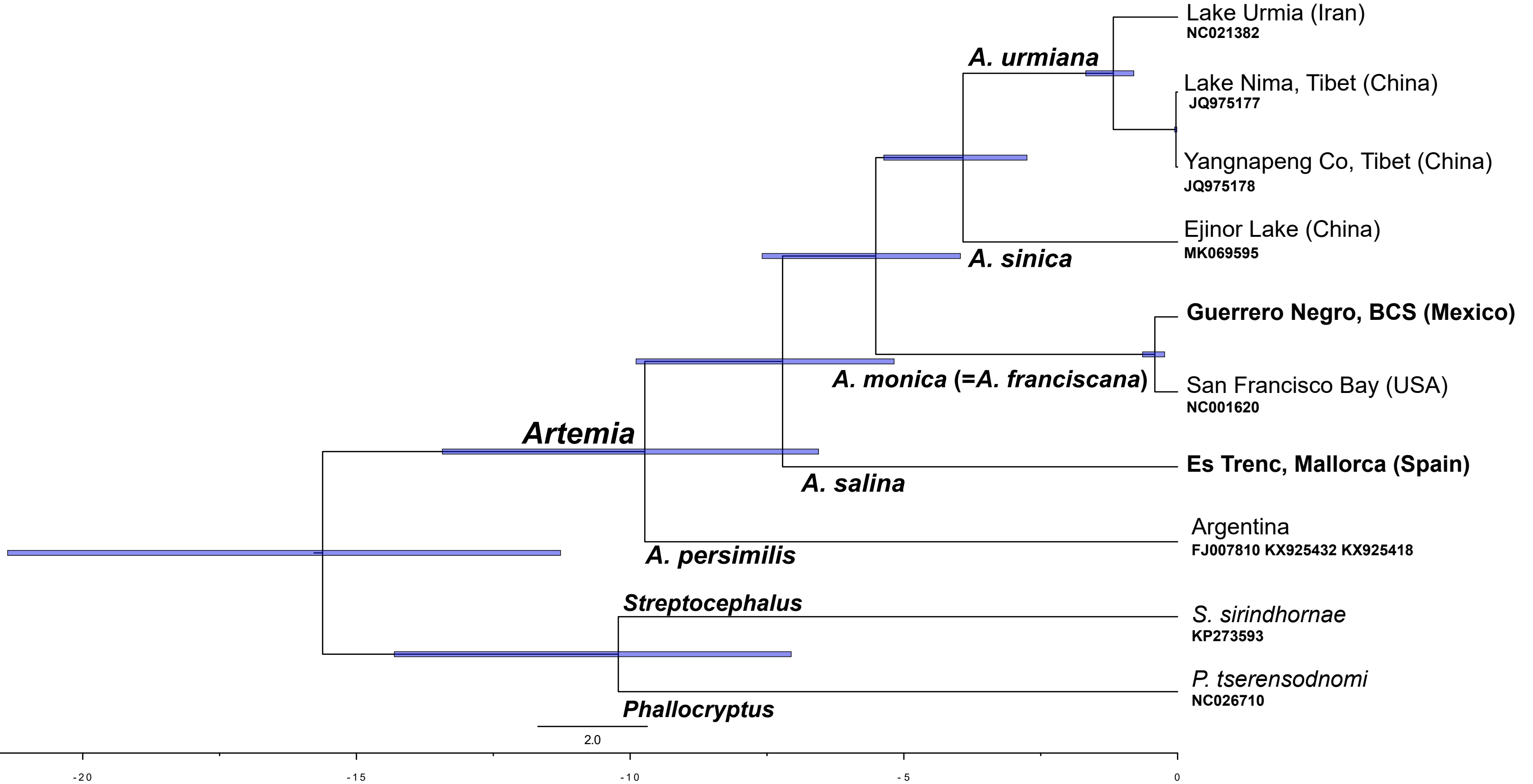

Supplement: Supplemental Information 3 — Chronogram showing lineage divergence times in Artemia obtained using BEAST following the third scenario hypothesis (Scheme 3). Time indicated in million years (Ma). Dark blue horizontal bars represent 95% HPD (High Posterior Density). A posterior probability value of 1 was obtained for all nodes. [file peerj-09-10865-s003.pdf]

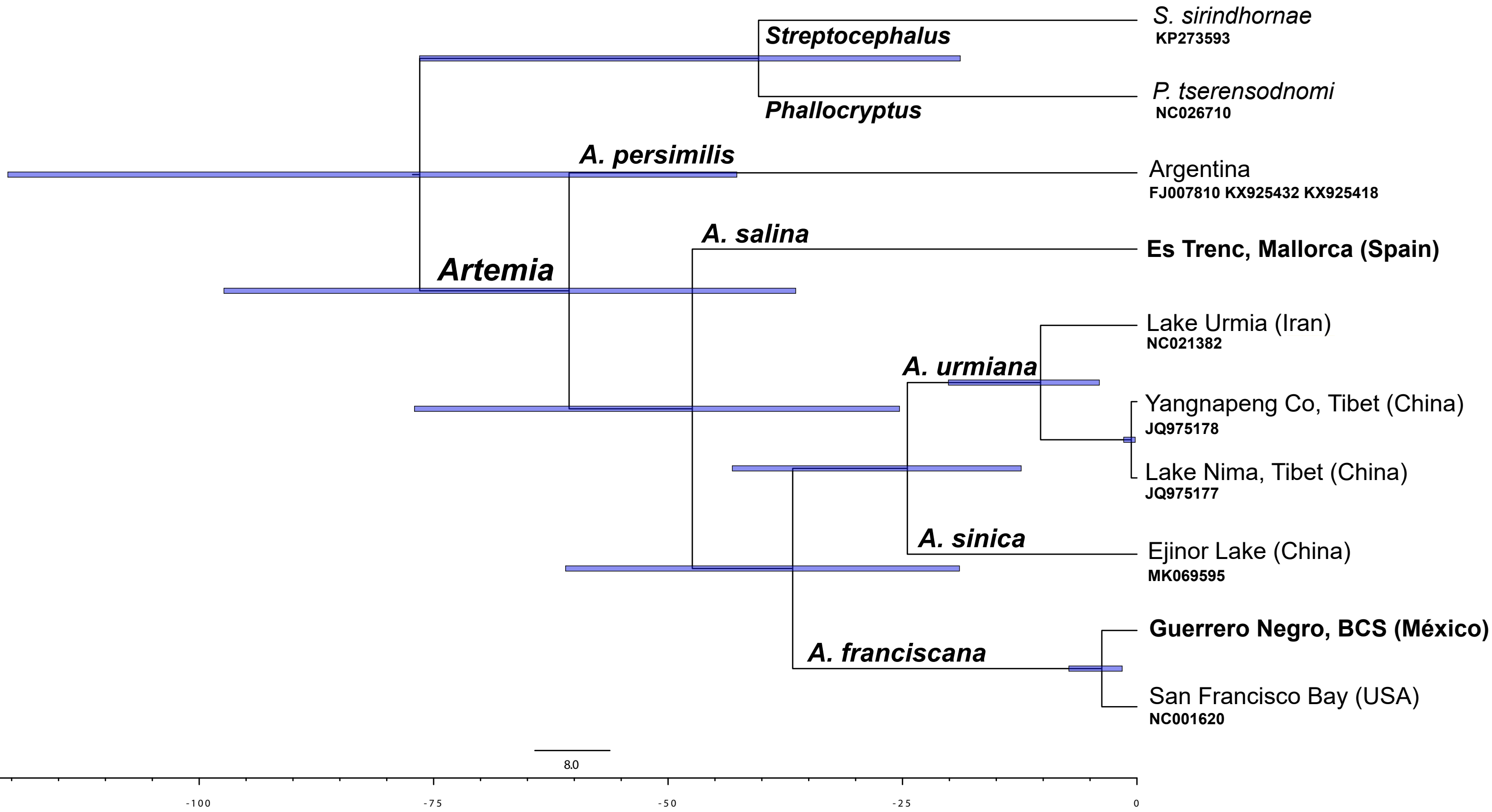

Supplement: Supplemental Information 4 — Chronogram showing lineage divergence times in Artemia obtained using BEAST following the fourth scenario hypothesis (Scheme 4). Time indicated in million years (Ma). Dark blue horizontal bars represent 95% HPD (High Posterior Density). A posterior probability value of 1 was obtained for all nodes. [file peerj-09-10865-s004.pdf]
