# Supplementary material for: Settling taxonomic and nomenclatural problems in brine shrimps, Artemia (Crustacea: Branchiopoda: Anostraca), by integrating mitogenomics, marker discordances and nomenclature rules
Source: PeerJ. 2021 Mar 10;9:e10865. doi: 10.7717/peerj.10865 (PMC7955675; doi:10.7717/peerj.10865)
Supplement: Supplemental Information 5 — Mitochondrial DNA contains 13 protein genes, two rRNA genes and 22 tRNA genes. Length, size, positions, start/stops codons (protein genes) and anticodons (tRNA) are specified per gene. DLP stands for D-loop and associated promoters. *TAA stop codon is completed by the addition of 3’ A residues to the mRNA. [file peerj-09-10865-s005.docx]

| **Gene** | **Strand** | **Location** | **Size** | **Anti Codon** | **Start Codon** | **Stop Codon** |
| --- | --- | --- | --- | --- | --- | --- |
| coxI | J | 1-1539 | 1539 |  | ATG | TAA |
| trnL2 | J | 1535-1598 | 64 | TAA |  |  |
| coxII | J | 1599-2285 | 687 |  | GTG | TAG |
| trnK | J | 2281-2344 | 64 | CTT |  |  |
| trnD | J | 2347-2407 | 61 | GTC |  |  |
| ATP8 | J | 2408-2569 | 162 |  | ATC | TAA |
| ATP6 | J | 2563-3222 | 660 |  | ATG | TAA |
| coxIII | J | 3222-4007 | 786 |  | ATG | TAA |
| trnG | J | 4009-4069 | 61 | TCC |  |  |
| NAD3 | J | 4070-4405 | 336 |  | ATT | TAG |
| trnA | J | 4429-4489 | 61 | TGC |  |  |
| trnR | J | 4490-4552 | 63 | TCG |  |  |
| trnN | J | 4553-4614 | 62 | GTT |  |  |
| trnS1 | J | 4615-4679 | 65 | GCT |  |  |
| trnE | J | 4679-4741 | 63 | TTC |  |  |
| trnF | N | 4740-4802 | 63 | GAA |  |  |
| NAD5 | N | 4803-6408 | 1606 |  | ATA | T(AA)* |
| trnH | N | 6424-6484 | 61 | GTG |  |  |
| NAD4 | N | 6485-7673 | 1189 |  | ATG | T(AA)* |
| NAD4L | N | 7666-7926 | 261 |  | ATC | TAA |
| trnT | J | 7928-7989 | 62 | TGT |  |  |
| trnP | N | 7990-8050 | 61 | TGG |  |  |
| NAD6 | J | 8053-8520 | 468 |  | GTG | TAG |
| cytB | J | 8538-9668 | 1131 |  | ATG | TAA |
| trnS2 | J | 9669-9735 | 67 | TGA |  |  |
| NAD1 | N | 9740-10636 | 897 |  | ATG | TAA |
| trnL1 | N | 10649-10710 | 62 | TAG |  |  |
| 16S | N | 10711-11857 | 1147 |  |  |  |
| trnV | N | 11858-11919 | 62 | TAC |  |  |
| 12S | N | 11920-12631 | 712 |  |  |  |
| trnM | J | 14105-14167 | 63 | CAT |  |  |
| NAD2 | J | 14168-15058 | 891 |  | ATG | TAA |
| trnW | J | 15057-15119 | 63 | TCA |  |  |
| trnI | N | 15139-15200 | 62 | GAT |  |  |
| trnQ | N | 15208-15273 | 66 | TTG |  |  |
| trnC | N | 15310-15370 | 61 | GCA |  |  |
| trnY | N | 15375-15436 | 62 | GTA |  |  |
| DLP |  | 12632-14104 | 1473 |  |  |  |
