# Supplementary material for: Settling taxonomic and nomenclatural problems in brine shrimps, Artemia (Crustacea: Branchiopoda: Anostraca), by integrating mitogenomics, marker discordances and nomenclature rules
Source: PeerJ. 2021 Mar 10;9:e10865. doi: 10.7717/peerj.10865 (PMC7955675; doi:10.7717/peerj.10865)
Supplement: Supplemental Information 6 — Annotations of the mitogenome content of Artemia franciscana. Mitochondrial DNA contains 13 protein-coding genes, two rRNA genes and 22 tRNA genes. Length, size, positions, start/stops codons (protein genes) and anticodons (tRNA) are specified per gene. DLP stands for D-loop and associated promoters. *TAA stop codon is completed by the addition of 3’ A residues to the mRNA. [file peerj-09-10865-s006.docx]

| **Gene** | **Strand** | **Location** | **Size** | **Anti Codon** | **Start Codon** | **Stop Codon** |
| --- | --- | --- | --- | --- | --- | --- |
| coxI | J | 1-1539 | 1539 |  | ATG | TAA |
| trnL2 | J | 1535-1598 | 64 | TAA |  |  |
| coxII | J | 1599-2285 | 687 |  | GTG | TAG |
| trnK | J | 2281-2344 | 64 | CTT |  |  |
| trnD | J | 2348-2409 | 62 | GTC |  |  |
| ATP8 | J | 2410-2571 | 162 |  | ATT | TAG |
| ATP6 | J | 2565-3224 | 660 |  | ATG | TAA |
| coxIII | J | 3224-4009 | 786 |  | ATG | TAG |
| trnG | J | 4011-4071 | 61 | TCC |  |  |
| NAD3 | J | 4072-4407 | 336 |  | ATT | TAA |
| trnA | J | 4437-4498 | 62 | TGC |  |  |
| trnR | J | 4499-4562 | 64 | TCG |  |  |
| trnN | J | 4563-4625 | 63 | GTT |  |  |
| trnS1 | J | 4626-4690 | 65 | GCT |  |  |
| trnE | J | 4690-4753 | 64 | TTC |  |  |
| trnF | N | 4752-4813 | 62 | GAA |  |  |
| NAD5 | N | 4815-6411 | 1597 |  | ATC | T(AA)* |
| trnH | N | 6435-6496 | 62 | GTG |  |  |
| NAD4 | N | 6497-7685 | 1189 |  | ATG | T(AA)* |
| NAD4L | N | 7678-7938 | 261 |  | ATC | TAA |
| trnT | J | 7940-8001 | 62 | TGT |  |  |
| trnP | N | 8002-8062 | 61 | TGG |  |  |
| NAD6 | J | 8065-8532 | 468 |  | ATC | TAG |
| cytB | J | 8558-9688 | 1131 |  | ATG | TAG |
| trnS2 | J | 9692-9758 | 67 | TGA |  |  |
| NAD1 | N | 9765-10661 | 897 |  | ATG | TAA |
| trnL1 | N | 10674-10735 | 62 | TAG |  |  |
| 16S | N | 10736-11890 | 1155 |  |  |  |
| trnV | N | 11891-11953 | 63 | TAC |  |  |
| 12S | N | 11954-12666 | 703 |  |  |  |
| trnM | J | 14483-14545 | 63 | CAT |  |  |
| NAD2 | J | 14546-15436 | 891 |  | ATG | TAA |
| trnW | J | 15435-15497 | 63 | TCA |  |  |
| trnI | N | 15515-15576 | 62 | GAT |  |  |
| trnQ | N | 15585-15650 | 66 | TTG |  |  |
| trnC | N | 15689-15751 | 63 | GCA |  |  |
| trnY | N | 15764-15825 | 62 | GTA |  |  |
| DLP |  | 12667-14482 | 1816 |  |  |  |
