# Supplementary material for: Settling taxonomic and nomenclatural problems in brine shrimps, Artemia (Crustacea: Branchiopoda: Anostraca), by integrating mitogenomics, marker discordances and nomenclature rules
Source: PeerJ. 2021 Mar 10;9:e10865. doi: 10.7717/peerj.10865 (PMC7955675; doi:10.7717/peerj.10865)
Supplement: Supplemental Information 7 [file peerj-09-10865-s007.docx]

| **Subset** | **Genes included** | **Best estimated model** |
| --- | --- | --- |
| 1 | ATP6, ATP8 | MTART+G |
| 2 | Cox1, Cox2, Cox3 | MTART+I+G |
| 3 | CytB | MTMAM+G |
| 4 | NAD1, NAD2, NAD3, NAD4, NAD4L, NAD5, NAD6 | MTREV+G+F |
| 5 | 12S, 16S | HKY+ G |
